# Mechanistic and Life-Cycle Framework for Green Nanomaterials in Atmospheric Water Harvesting

**DOI:** 10.3390/nano16070433

**Published:** 2026-03-31

**Authors:** Noor Al-Sadeq, Johar Amin Ahmed Abdullah, Alberto Romero, Víctor M. Perez-Puyana

**Affiliations:** 1 Departamento de Ingeniería Química, Facultad de Química, Universidad de Sevilla, 41012 Sevilla, Spain; jabdullah@us.es; 2 Laboratorio de Rayos-X, Centro de Investigación, Tecnología e Innovación de la Universidad de Sevilla, CITIUS, Universidad de Sevilla, 41012 Sevilla, Spain; 3 Departamento de Ingeniería y Ciencia de los Materiales y del Transporte, Universidad de Sevilla, 41092 Sevilla, Spain; vperez11@us.es

**Keywords:** atmospheric water harvesting (AWH), thermodynamics, green nanotechnology, life-cycle assessment, sustainable nanomaterials, adsorption–desorption mechanisms

## Abstract

Atmospheric water harvesting (AWH) has been recognized as a promising technology to address global freshwater scarcity in a decentralized manner. Nevertheless, conventional AWH sorbents are often associated with high energy consumption, toxic synthesis procedures, and short operational lifetimes. To address such limitations, a comprehensive review paper develops a unified framework to bridge the gap between nanoscale material properties, such as synthesis routes, structural architecture, and adsorption thermodynamics, and macro-scale environmental and economic performance. This review paper rigorously examines emerging nanomaterials such as metal–organic frameworks (MOFs), covalent organic frameworks (COFs), mesoporous metal oxides, and graphene oxide derivatives. By highlighting benchmark materials such as MOF-303 and passive solar-regenerated COF-ok, the review paper emphasizes the advantages of bio-assisted “green” synthesis routes. Crucially, this review extends beyond traditional water uptake figures and incorporates comprehensive Techno-Economic Assessments (TEA) and Life-Cycle Assessments (LCA). It examines various real-world influences, such as cumulative energy demand, levelized costs of water, and ton-scale manufacturing viability, to name a few. This report bridges atomic-level mechanics with industrial economics, and by so doing, offers design criteria to guide researchers in crafting a new generation of sustainable AWH infrastructure, with a focus on hierarchical pores, surface chemistry, and photothermal design.

## 1. Introduction

Recently, reliable freshwater availability is a key global challenge in arid and semi-arid regions where traditional supplies are increasingly stretched due to population growth, climate variability, and unequal resource distribution [1,2,3]. Compared to centralized desalination and long-distance water delivery, atmospheric water harvesting (AWH) offers a promising decentralized method of extracting water from ambient air, especially in distant or resource-constrained situations [4,5,6]. The Earth’s atmosphere contains an estimated 13 trillion tons of water vapor. This is a vast, ubiquitous, and continually replenished supply. Collecting this vapor in a location-independent manner requires little in the way of macroscopic infrastructure [7].

Historically, AWH technologies have employed direct refrigeration principles in which ambient air is cooled to a temperature lower than its dew point. Although successful in humid and tropical environments, the use of condensers in arid environments (RH < 30%) is marred by catastrophic thermodynamic efficiency losses, often necessitating specific energy consumption (SEC) values in excess of 1.0 kW∙h∙L^−1^ in order to obtain minimal quantities of water [8]. To address the aforementioned thermodynamic limitations, recent breakthroughs in AWH technology have resoundingly moved towards sorption-based AWH technology, which involves the application of advanced nanostructured materials exhibiting exceptionally high water-vapor affinity, thereby permitting the spontaneous absorption of water vapor at ambient temperatures and depressed humidity levels, followed by low-enthalpy regeneration using natural sunlight or waste heat.

The development of nanostructured materials with enhanced water-vapor affinity is largely responsible for recent advancements in sorbent-based AWH technology. Because of their adjustable porosity, surface chemistry, and adsorption behavior, metal oxides, porous silica materials, graphene-based composites, and metal–organic frameworks (MOFs) have been the subject of several investigations [9,10,11,12]. Among these material types, MOFs have drawn special attention due to their distinctive pore topologies, which allow abrupt shifts in water uptake at particular relative humidity ranges, mainly significant in dry weather. For instance, metal–organic frameworks (MOFs) can be rationally designed to demonstrate abrupt, step-wise water uptake at very specific relative humidity thresholds. The zirconium-based MOF-801 (Zr-fumarate) contains hydrophilic Zr-oxo clusters and exhibits a sharp water-uptake step around 20% RH, making it suitable for low-humidity atmospheric water harvesting. In an arid-climate device demonstration in Tempe, Arizona, it was reported to deliver over 0.25 L of water per kg of MOF in a single daily cycle [13]. The aluminum-based MOF-303 (Al-pyrazolate) has been rationally designed with highly accessible hydrogen bonding sites in its pyrazolate linkers, allowing it to demonstrate exceptionally rapid adsorption/desorption kinetics [14].

Despite these unprecedented material-level advancements, scaling up from proof-of-concept laboratory synthesis to globally scalable industrial infrastructure remains a challenge. While high gravimetric water capacity is not sufficient for a material-level assessment of its practicality, its performance is largely dictated by its regeneration temperature, cyclic stability, resistance to biological fouling, and life-cycle persistence [15,16,17]. In addition, the conventional synthesis of the best performing nanoporous materials often requires the use of hazardous organic solvents, extreme temperatures, and complex purification schemes, which undermine the fundamental sustainability goals of AWH technology [18].

High gravimetric water intake by itself cannot ensure practical feasibility. Pore size distribution, surface functional groups, and capillary condensation thresholds are examples of nanostructural properties that regulate water sorption behavior and are greatly influenced by the synthesis process [19,20,21]. Long-term performance and operational efficiency are significantly influenced by regeneration temperature, cycling stability, and susceptibility to deterioration or fouling [15,22,23,24]. Throughout the material life-cycle, these material-level traits are intimately linked to more general system-level elements like embodied energy, solvent use, and environmental effect. However, a lot of recent evaluations only consider adsorption processes and sustainability metrics, which obscures significant trade-offs that are pertinent to practical implementation [24,25,26].

In response to such holistic challenges, “green nanotechnology” has emerged as a revolutionary science. By focusing on “benign solvents,” “biogenic precursors,” and “ambient-pressure synthesis pathways,” green nanotechnology promises to “separate the capabilities of advanced materials from toxic and energy-intensive manufacturing processes” [27,28,29,30]. Yet, holistic evaluations of such mechanistic effects of green nanotechnology synthesis pathways on the thermodynamic efficiency of AWH systems remain limited [31,32,33].

This review focused on the mechanistic and life-cycle framework for green nanomaterials in atmospheric water harvesting. It systematically discussed the relation between synthesis techniques, structural characteristics, water adsorption and desorption mechanisms, and overall sustainability of the AWH materials, more specifically, metal oxides, silica-based systems, graphene derivatives, and MOFs. The review considers how surface chemistry, pore architecture, and regeneration techniques affect material performance in real-world humidity conditions. Additionally, this review discussed the potential and challenges for large-scale development by combining life-cycle issues, including energy consumption, solvent use, regeneration temperature, and end-of-life management. Finally, it presents a comparison framework to evaluate new AWH materials and direct the creation of effective and ecologically friendly designs.

## 2. Fundamentals of Atmospheric Water Harvesting: Mechanistic Considerations

The term “atmospheric water harvesting” (AWH) refers to a variety of technologies intended to collect and transform atmospheric moisture into liquid water. These technologies can be broadly divided into two categories: natural and engineering (man-made) techniques. Unique physical mechanisms control each and show varying degrees of applicability based on infrastructural needs, energy availability, geographic restrictions, and climate. In order to provide the mechanistic foundation needed to assess material-level design decisions covered in the following parts, these principles are presented here. Table 1 presents a comparative summary of these methods, along with examples of commercial applications [4].

### 2.1. Principles of Atmospheric Water Harvesting

Water vapor, an abundant but intermittent source of freshwater, is extracted from the atmosphere by the AWH process. The most common methods of AWH include condensation, adsorption, and fog collection. Condensation is achieved by cooling the atmosphere below the dew point. Adsorption is defined as the adhesion of water vapor to the surface of hygroscopic materials by chemical or physical interactions. Absorption is defined as the penetration of water vapor into the bulk of the adsorbent. Fog collection is carried out on wet or rough surfaces [19,20,21]. All these processes depend on atmospheric temperature and relative humidity.

Recently, sorption-based atmospheric water harvesting has gained significant attention due to its potential to use low-grade thermal or solar energy. The thermodynamic properties of sorption-based water harvesting include surface free energy, enthalpy of regeneration, and chemical potential of water vapor. For example, MOFs can be rationally designed at the molecular level to maximize water adsorption capacity at low relative humidity. Zeolites and silica gel are preferred for their ease of handling. Adsorption-based water harvesting is sensitive to nanoscale pore structure and surface chemistry. Surface properties play an important role in water adsorption capacity, desorption energy, and cyclic operation.

### 2.2. Technological Approaches to Atmospheric Water Harvesting

As mentioned earlier, the principles of AWH include condensation, adsorption, and fog collection [19]. The condensation principle cools the air to a temperature lower than its dew point, hence converting water vapor to water droplets. The system can produce large volumes of water in a very short time but can consume a lot of power. At optimum humidity, it requires about 0.3 to 1.0 kWh of power to produce 1.0 L of water [35]. The efficiency of the system reduces under low humidity conditions because it requires a lot of power to produce a small volume of water. The adsorption principle has its advantages and disadvantages. The system can be operated under low-grade heat, hence reducing power consumption. The efficiency of the system increases under low humidity conditions. The water output of the system is lower than that of condensation. The fog collection principle has the lowest power consumption. The system can be operated under wind power. The system can only be used in regions where fog occurs. The condensation principle can be used where large volumes of water output and power consumption can be accommodated. The adsorption principle can be used in dry to semi-dry regions where low-grade heat can be utilized. The fog collection principle can be used in regions where fog occurs.

The adsorption-based AWH systems absorb water vapor from the ambient air using hygroscopic salts, polymers, or porous materials. The water is then released by controlled heating. These systems are ideal for decentralized applications in arid environments as they use low-grade heat sources, such as solar energy and industrial waste heat. The operational efficiency of these systems is affected by equilibrium absorption, sorbent thermodynamics, regeneration temperature, and cycling stability.

Fog-collection AWH systems catch airborne droplets using textured meshes or fibers, and their efficiency is affected by a number of factors, including the size of the droplets, wind speed, mesh shape, and surface wettability. Integrating these systems with nanomaterials increases their efficiency due to the increase in the surface area, assisting with capillary condensation [14]. Consequently, this approach allows the photothermal or hybrid regeneration. As a result, in order to make atmospheric water harvesting more resilient, sustainable, and effective, materials design is now just as crucial as system architecture.

### 2.3. Key Parameters Influencing AWH Performance

The AWH system performance is affected by sorption efficiency, regeneration energy consumption, and long-term stability through thermodynamic, material, and operational parameters. The key parameters and their typical ranges that affected the AWH system performance documented in the literature across different operating situations are tabulated in Table 1. These factors depend on one another; for instance, while materials designed for low regeneration temperatures may exhibit decreased cycling stability, increasing sorption capacity through increased water–surface interactions can result in higher regeneration enthalpy. Therefore, one can conclude that it is insufficient to optimize particular indicators separately. For effective material selection and system design, a detailed evaluation of adsorption thermodynamics, regeneration energy, durability, and operating conditions is required.

### 2.4. Limitations and Transition Toward Material-Centric Design

Despite being technically feasible, conventional AWH approaches have significant limitations that prohibit widespread implementation. Since the mechanical condensation systems are affected by constant electrical input and refrigeration devices, they need a lot of energy at low levels of humidity. However, after several cycles of desiccation, the desiccants made of salt will lose their punch due to leaching and corrosion. When the salt desiccants absorb moisture, they will be weakened due to chemical reactions with the minerals or air components in the environment. This weakening is corrosion. The active component will be depleted from the desiccant due to the dissolution of the salt by the moisture during the renewal or adsorption processes. On the other hand, the desiccants made of hydrogel and polymers will experience slower kinetics and temperature stability. The high density of these materials will result in slower kinetics of water diffusion, delaying the desiccation process. These desiccants will experience reduced stability when used at temperatures away from the optimal range, further deteriorating their stability. All these limitations will result in a higher energy consume, loss of materials, and more maintenance, as they relate to mass transport, thermodynamics, and materials stability [36,37,38].

This drive towards material-centered design is rooted in our awareness of our limitations. For instance, in the case of metal–organic frameworks, it is possible to fine-tune their nanometer-scale pore structure with great precision, striving for regeneration at lower temperatures and maximizing water uptake. Besides, there is a move towards greener synthesis techniques in the design of hybrid and nanostructured sorbents, making it environmentally friendly compared to traditional synthesis techniques. The aforementioned techniques intentionally fine-tune surface chemistry, regeneration thermodynamics, and nanometer-scale architecture in order to take the performance of AWH systems beyond optimization. This is in contrast to simply maximizing adsorption capacity. This balance is at the heart of the mechanistic thermodynamic analysis presented in Section 2.5.

### 2.5. Mechanistic Thermodynamics of Adsorption–Desorption

Thermodynamic driving forces that link temperature, surface energy, and vapor pressure regulate water vapor capture in AWH systems. The design and performance analysis of adsorption-based AWH are based on the idea that adsorption becomes beneficial in nanostructured materials when the chemical potential of water vapor exceeds that of the adsorbed phase, resulting in spontaneous uptake driven by a drop in free energy [4,6]. The surface chemistry and pore size distribution of the AWH systems dictate the water absorption isotherm approach and the relative humidity (RH) threshold for fast adsorption in porous sorbents. For instance, adsorbents having micropores (<2 nm) and mesopores (2–50 nm) are known to encourage capillary condensation at particular RH values; for hydrophilic materials, fast water uptake usually begins below 30% RH. For pore sizes of 0.5–2 nm, water uptake steps in certain MOFs take place in the 10–25% RH range. At RH levels as low as 20–30%, silica gels, which typically have a pore size distribution between 2 and 20 nm, show notable adsorption. Capillary condensation starts at decreasing relative humidity when pore diameters are lowered to the micro- or mesoporous area. MOFs and silica-based materials designed for arid and semi-arid settings commonly use this technique [15,17].

These thermodynamic concepts have practical implications in the real world, especially in the way in which the water harvesting system based on sorption actually adsorbs water. For instance, in the case of MOF-801 (Zr-fumarate), this material has hydrophilic Zr-oxo clusters and has a pore diameter of about 0.6 nm. At room temperature, the material shows a step in water adsorption at about 20–25% relative humidity. This indicates that the material can function even in drier environments. This is because of the high hydrophilicity of the material, which allows the water molecules and the Zr-oxo clusters to have a high attraction for each other. This reduces the activation energy required for the adsorption of water molecules [25,28]. On the other hand, the MOF-303 (Al-pyrazolate) material has fast kinetics of adsorption and desorption and high reversibility. This is because of the presence of hydrogen bonding sites. This material has many advantages over other materials, such as MOF-801. For instance, the presence of many hydrogen bonding sites allows the material to have fast kinetics of adsorption and desorption. Moreover, the material has a high lifespan. At higher relative humidity of about 30–35%, the material starts adsorbing water strongly [26]. At this high relative humidity, the material undergoes capillary condensation. This phenomenon depends on the size of the material and the hydroxyl density [15,17].

In order to break the cling of water on the surface and restore the adsorption capacity, you have to supply heat to the system. This is an enthalpy-driven desorption. The regeneration can be achieved using solar power, photothermal effects, or heating. The photothermal nanomaterials assist in cutting down the power necessary for regeneration by generating heat on the surface of the sorbent. This reduces heat loss because it directly heats the sorbent [6,29]. Adsorption-based atmospheric water harvesting materials need 1.2 to 2.5 MJ of power for every kilogram of water harvested. The advanced MOFs can achieve 1.3 MJ/kg H_2_O with solar power. The stability of the sorbent after a large number of cycles is important. The sorbents can retain greater than 90% of their initial capacity after 100 to 500 cycles. The sorption kinetics of these materials differ. The kinetics allow for full adsorption–desorption cycles within 30 to 90 min at room temperature [39]. Adsorption capacity increases with the adsorption capacity [29,33]. The higher the adsorption capacity, the higher the regeneration power. The higher regeneration power has a negative effect on capacity and cycle stability. The lower adsorption capacity has a positive effect on capacity because it reduces the regeneration power. The kinetics and cycle stability of the sorbent are important for adsorption-based water harvesting materials [25,33].

With regard to materials design, the controlled adjustment of surface chemistry, regeneration pathways, and pore structure is enabled by thermodynamic principles. The latter enables the reduction of energy consumption while maintaining high water uptake capacity and good cycling stability. From this mechanistic foundation, the following comparative study of green nanomaterials extends to the assessment of adsorption–desorption capacity and synthesis routes/life-cycle consequences [4,29].

**Figure 1:**
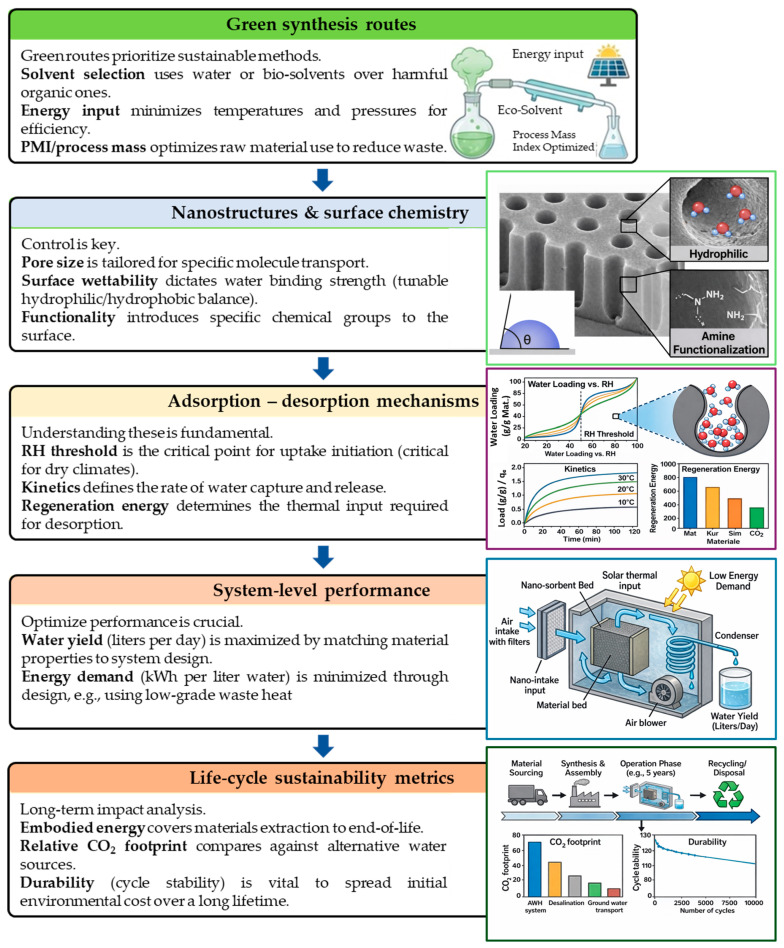
Conceptual roadmap integrating mechanistic adsorption–desorption principles with life-cycle sustainability considerations for nano-enabled atmospheric water harvesting systems [40,41,42].

In summary, the mechanistic life-cycle approach of this study has five pillars: (1) green synthesis routes that influence the chemical and structural properties of nanomaterials, (2) surface chemistry and nanostructure design that control adsorption–desorption kinetics and capacity, (3) regeneration routes that influence energy consumption and material stability, (4) water yield and system performance, and (5) life-cycle sustainability considerations such as energy efficiency, recyclability, and environmental footprint. As depicted in Figure 1, these components form an integrated framework for the assessment and comparison of cutting-edge AWH materials. The following comparative study maintains its internal consistency based on this mechanistic life-cycle approach.

## 3. Green Nanotechnology: Principles and Applications

Conventionally, the fabrication of such sophisticated nanoporous materials has been achieved by solvothermal synthesis routes that involve the use of hazardous and environmentally unfriendly organic solvents with high boiling points (e.g., N,N-Dimethylformamide, abbreviated as DMF), high pressures, and high temperature reaction conditions. These routes produce a tremendous amount of hazardous chemical wastes and an immense “embodied energy penalty,” which is fundamentally contradictory to the philosophy of sustainable and “decentralized water harvesting” [43].

Green nanotechnology combines green chemistry with sustainable engineering to design nanomaterials that minimize environmental impacts from the synthesis, application, and disposal phases [44]. In addition, green synthesis methods employ green chemistry to design the synthesis routes for the nanomaterials. This is the reason for the considerable interest in these routes for the design of sorbent nanomaterials for AWH applications due to their influence on the structure and regeneration of the sorbent material, as well as their environmental sustainability. Moreover, green synthesis methods reduce the environmental impact of adsorption-based AWH by varying the characteristics of the resultant nanostructures. For instance, hydrophilic functional groups and hierarchical porosity are often introduced by bio-derived reducing and capping agents, which can alter water absorption isotherms and improve adsorption at lower relative humidity. These advantages, however, are not always seen and heavily rely on the chemistry of the precursor, the processing environment, and the post-synthesis procedures. Therefore, rather than assuming inherent performance benefits, a critical study of each system is required.

### 3.1. Principles and Green Synthesis of Nanomaterials

Throughout the nanomaterial life-cycle, green nanotechnology uses fundamental concepts of green chemistry, such as energy efficiency, the use of non-toxic reagents, reliance on renewable resources, and waste reduction [44,45]. Because material sustainability directly affects environmental performance at the system level, these concepts are especially important for water treatment and AWH applications. Compared to traditional solvothermal or chemical precipitation techniques, several life-cycle studies have shown decreases in embodied energy and related emissions. However, the synthesis method, material size, and solvent recovery efficiency all have a significant impact on how much these reductions occur [44,45,46].

By using bio-assisted synthesis techniques, surface functionalities that improve hydrophilicity and water–surface interactions can be added, which may improve adsorption kinetics and reduce effective regeneration temperatures. However, the long-term robustness and cycle stability of these surface alterations may be impacted by frequent humid–dry cycling. As a result, green synthesis methods should be evaluated for their operational stability in AWH systems as well as their mechanistic implications on adsorption and desorption behavior.

#### 3.1.1. Plant- and Agricultural-Waste-Based Synthesis

Bioactive compounds including polyphenols, flavonoids, organic acids, and sugars that act as reducing, chelating, and stabilizing agents in the creation of nanoparticles can be found in large quantities and at low cost in plant extracts and agricultural residues [46,47]. For instance, *Citrus sinensis* (orange peel) extracts allow for the aqueous production of silver nanoparticles at temperatures below 100 °C without the need for extra surfactants, producing monodisperse particles with strong surface reactivity [47]. Similarly, under mild conditions, *Phoenix dactylifera* (date palm) leaf extracts promote the synthesis of homogeneous iron oxide nanoparticles (2–30 nm), leading to materials with improved colloidal stability [48].

Mesoporous SiO_2_ and ZnO nanostructures can also be produced from silica-rich agricultural wastes such as rice husk ash and banana peels [49,50,51]. ZnO generated from banana peels has a lot of surface hydroxyl groups and intrinsic photocatalytic activity, which can encourage antibacterial activity and solar-assisted regeneration [51,52]. Biomass- or waste-derived silica materials prepared by sol-gel or templating approaches can exhibit high specific surface area; for example, MCM-41-type mesoporous silica has been reported with a BET surface area of about 938.6 m^2^ g^−1^, although surface area varies strongly with precursor source, templating route, and final composition [50,53,54].

Based on regeneration conditions and long-term stability, hybrid materials that combine bio-derived SiO_2_ with ZnO or other metal oxides form hierarchical architectures that couple high surface area with photothermal responsiveness, potentially offering advantages for adsorption–desorption cycling efficiency in AWH systems [53,55].

#### 3.1.2. Microbial and Biogenic Routes

Microbial synthesis reduces the need for hazardous chemicals or high-energy processing by utilizing the metabolic activity of bacteria, fungi, and yeasts to reduce and stabilize metal ions under near-ambient conditions (30–37 °C, atmospheric pressure). For instance, extracellular proteins and polysaccharides act as natural capping agents, preventing aggregation and enhancing colloidal stability. On the other hand, enzymes such as nitrate reductase and NADH-dependent oxidoreductases catalyze metal-ion reduction [56,57]. These biosynthetic pathways yield nanoparticles with a size range of 2.5–70 nm, forming stable dispersions in AWH systems under humid conditions and exhibiting antibacterial properties [58,59]. Interestingly, microbial synthesis is better viewed as an auxiliary method rather than a widely applicable production technique for AWH sorbents, given persistent issues with scalability, batch-to-batch variability, and long-term durability across repeated adsorption–desorption cycles.

#### 3.1.3. Environmental Benefits and Relevance to AWH

Generally, green synthesis approaches, such as plant-based, waste-derived, and microbial processes, reduce emissions, energy consumption, and solvent toxicity, compared to the conventional chemical or solvothermal methods [47,48,49,50,51,52,53]. These techniques often result in water-synthesized metal–organic frameworks (MOFs), Fe_2_O_3_/Fe_3_O_4_, ZnO, TiO_2_, SiO_2_, and other nanoparticles with improved hydrophilicity and accessible surface area. These characteristics can help with adsorption at moderate-to-low humidity levels and lower the temperature needed for regeneration. The success of these techniques relies on the surface functionality of the materials, how they perform during successive cycles, and the engineered pore structures of the materials. These factors explain the need for us to consider green synthesis techniques from the perspective of life-cycle analysis, rather than simply assuming them to be better by default. Green synthesis provides a useful, albeit qualified, approach for reconciling nanoscale materials design with the principles of sustainability in AWH. The subsequent sections will further discuss this point.

### 3.2. Life-Cycle Assessment (LCA) and the “Greenwashing” Paradigm

Although plant-based synthesis seems to be environmentally friendly in its own right, for true environmental sustainability to be achieved, validation through rigorous cradle-to-gate Life-cycle Assessment (LCA) in accordance with ISO 14040/44 is necessary [60]. The literature indicates that replacement of a chemical reductant with a biological extract does not necessarily imply a more sustainable process. If the green synthesis route requires sustained electrical heating to sustain reaction temperature levels, or requires massive volumes of ethanol for purification steps, the Cumulative Energy Demand (CED) and aquatic ecotoxicity potential can quickly escalate beyond that of traditional chemical precipitation routes. For instance, in the OpenLCA modeling of the synthesis of titanium dioxide (TiO_2_) nanoparticles, it was found that although the use of *Cymbopogon citratus* (lemon grass) extracts reduced the direct chemical toxicity and greenhouse gas emissions considerably compared to the traditional chloride route, the electrical energy required for the stirring and centrifugation steps was the primary contributor to the environmental impact potential [28]. Thus, for green nanomaterials to show a clear environmental advantage, the bio-assisted chemical route needs to be combined with scalable and high-yielding continuous flow or mechanochemical synthesis routes [60].

### 3.3. Green Nanoparticles Replacing Traditional Desiccants

In addition to synthesis, green nanomaterials also physically resolve the operational failures associated with traditional AWH desiccants. In humid and wet–dry cycle conditions, silica gel and polymeric hydrogel desiccants are highly prone to biological fouling. Airborne microorganisms accumulate on the moist and porous structure, creating an impermeable biofilm that disrupts the kinetics, reduces water yields, and compromises the quality of the extracted water.

Green nanotechnology addresses this issue through the structural integration of biogenic antimicrobial agents into the sorbent matrix. Silver nanoparticles (AgNPs), synthesized using plant extracts, and green-synthesized zinc oxide (ZnO) nanoparticles are known to possess potent localized antimicrobial activity through the release of metal ions and ROS. The addition of as low as 0.15 wt.% biogenic ZnO and SiO_2_ nanoparticles to chitosan biopolymer aerogel has been reported to completely inhibit microbial colonization after extensive cycling [61]. Additionally, the nanoparticles impart significant mechanical strength to the hydrogel walls, thereby preventing structural failure during fast thermal desorption, and improving the efficiency of water harvesting from the sorbent matrix, as compared to undoped matrices [61].

### 3.4. Applications of Green Nanomaterials in Water Treatment: Implications for AWH Systems

Green nanomaterials have received considerable attention for their application in the treatment of both industrial and municipal water supplies, considering their ecofriendliness, high efficiency, and surface chemistry [62,63,64,65]. The characteristics of green nanomaterials provide insight into the durability, resistance to fouling, regeneration strategies, and overall performance, all of which are important considerations for the application of adsorption-based AWH technology. The application of nanomaterials that are exposed to wet–dry cycles in AWH technology would particularly benefit from the learnings from wastewater treatment concerning the operation of wet–dry cycles, the reusability of the nanomaterials, and their regeneration at low costs.

Water treatment processes are often associated with the application of large amounts of chemicals, the generation of sludges, and the use of considerable amounts of energy. Nanotechnologies provide the opportunity for the application of magnetic nanoparticles for separation, bio-derived nanoadsorbents for the capture of large amounts of contaminants, and nanocomposite membranes for resistance against fouling and longer lifetimes [62,63,64,65,66,67,68,69,70,71,72]. The characteristics of nanotechnologies, including their reusability, resistance to fouling, and the possibility for low-cost regeneration, are particularly desirable for the application of nanomaterials for AWH technology, considering the requirement for durability over multiple cycles of adsorption–desorption.

By enabling efficient separation and reuse in coagulation–flocculation, magnetic iron oxide nanoparticles lower sludge generation and chemical input. In a similar vein, magnetic or hybrid AWH sorbents reduce material loss during the cycle and aid in recovery. Surface functionalization and hierarchical porosity enhance absorption efficiency while preserving regeneration viability in adsorption processes, as demonstrated by green-synthesized oxides and bio-derived nanoadsorbents. Additionally, material lifetime and fouling minimization are given top priority in water filtration nanocomposite membranes. These elements are equally important for sorbent-based AWH systems exposed to biological contaminants, moisture, and dust.

Table 2 provides a comparative summary of representative water treatment applications of green nanomaterials, highlighting key performance characteristics that are transferable to AWH system design, such as surface chemistry control, regeneration routes, durability, and environmental effect. This analysis focuses on mechanisms and material features that guide the selection, optimization, and life-cycle assessment of nanomaterials for atmospheric water harvesting rather than providing a comprehensive comparison of wastewater methods. In this regard, water treatment applications provide a useful foundation for comprehending how green nanomaterials might be designed to balance long-term stability, regeneration energy, and adsorption efficacy in sustainable AWH systems.

### 3.5. Relevance to Contemporary Water-Management Challenges

Green nanomaterials are being investigated more and more as enabling elements for AWH and water treatment systems that seek to lower energy consumption in comparison to traditional centralized technologies. Under favorable operating conditions, porous membranes, aerogels, and sorbent-based materials can function under ambient pressure, gravity-driven flow, or low-grade thermal input. Several case studies report significantly lower energy consumption when compared to thermal desalination or reverse osmosis [62,64]. Point-of-use water purification and small-scale AWH devices are examples of decentralized deployment in rural, emergency, and off-grid settings that are supported by such material-level efficiencies and modular system design [65].

The use of benign solvents, renewable feedstocks, and lower-temperature processing are the main ways that certain green synthesis routes can lower embodied carbon and processing-related emissions in comparison to conventional nanomaterial production, according to life-cycle assessment studies carried out in compliance with ISO 14040 frameworks [73]. Depending on system limits, scale, and assumptions, reported reductions vary greatly, highlighting the significance of context-specific evaluation over generalized claims. Concurrently, bio-derived materials like biogenic nanoscale zero-valent iron (nZVI) are being evaluated through pilot-scale deployments by water utilities in North America and Europe, and their inclusion in environmental registries is growing [68,69].

Recent developments show how green nanomaterials are becoming more and more relevant for solving current water management issues. Nonetheless, a thorough evaluation of performance, durability, and life-cycle implications under actual working conditions is still crucial. In the context of atmospheric water harvesting (AWH), these factors are essential for converting laboratory-scale inventions into scalable and ecologically viable water-harvesting technology.

### 3.6. Applications of Green Nanomaterials in Atmospheric Water-Harvesting Systems

Because of their adaptable surface chemistry, adjustable pore topologies, and compatibility with low-energy regeneration processes, green-synthesized nanomaterials are increasingly being investigated as functional elements in AWH systems [70,74]. Different material classes offer distinct advantages based on the requirements for durability, regeneration methods, and environmental humidity. Sorbent qualities must therefore be customized to specific operational scenarios rather than relying on general performance standards.

Iron oxide nanoparticles (Fe_2_O_3_/Fe_3_O_4_) are commonly integrated with AWH sorbents to enhance the thermal conductivity and facilitate magnetic operation, through reducing the material loss during operation cycles by encouraging partial sorbent recovery and improving heat transmission during solar-driven or photothermal regeneration processes [48,75]. However, the overall water uptake is affected by the nanoparticles’ dispersion, loading percentage, and interactions with the host sorbent matrix.

ZnO-based nanostructures are naturally hydrophilic and antimicrobial, and thus, can reduce the biofouling in humid operating settings [51,58]. These characteristics are especially important for AWH systems that are exposed to dust and airborne microbes. However, it is important to carefully assess ZnO’s long-term durability during several adsorption–desorption cycles as well as its susceptibility to photocorrosion in the presence of sunshine when using it as an active sorbent component.

TiO_2_ nanostructures are commonly utilized for photocatalytic self-cleaning, which breaks down organic contaminants and partially renews surfaces when exposed to sunshine [63]. Instead of being the main water sorbent, TiO_2_ usually serves as an auxiliary component. Surface accessibility and composite design affect its impact on adsorption capacity. This feature improves the long-term functionality of outdoor atmospheric water collection systems.

Biomass-derived SiO_2_ aerogels and mesoporous silica materials represent an important class of environmentally sustainable sorbents for AWH due to their large specific surface area and variable pore size distribution [15,50,53]. Silica-based approaches are especially beneficial for dry and semi-arid environments, where capillary condensation can be encouraged at moderate to low relative humidity. However, for long-term application, the mechanical strength and resistance of the material to structural degradation are essential.

MOF-based sorbents, such as those derived from aqueous or low-temperature solvothermal approaches like MOF-801 and MOF-303, exhibit high water absorption capacity and lower carbon footprint compared to conventional solvothermal approaches [30,70]. These materials are appealing for low-humidity AWH applications because of their sharp humidity-dependent adsorption transitions. However, in order to combine performance with life-cycle impact, their practical implementation necessitates rigorous control of synthesis repeatability, stability under cycling, and regeneration conditions.

Collectively, these examples show how green nanomaterials can be customized to solve particular issues in AWH systems while simultaneously highlighting the fact that no single material class provides a universal solution. Therefore, a mechanistic and life-cycle-informed selection of nanomaterials that takes into account adsorption behavior, regeneration energy, durability, and environmental impact under practical operating conditions is necessary for effective AWH design.

### 3.7. Comparative Evaluation of Green Versus Conventional Synthesis Routes

**Figure 2:**
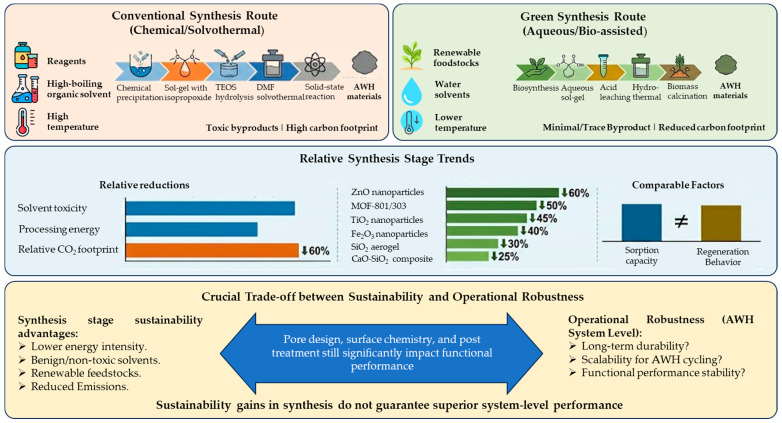
Comparative evolution of green versus conventional synthesis routes for AWH materials.

Comparative trends reported in the recent literature suggest that green synthesis routes can offer meaningful sustainability advantages for AWH materials when evaluated at the material-production stage (Table 3). Studies show that compared to traditional synthesis pathways, solvent toxicity, processing energy consumption, and related emissions are reduced across a variety of material classes. At the same time, sorption capacity and regeneration behavior are comparable within the range of reported experimental conditions. These trends are primarily attributed to the use of renewable feedstocks, aqueous or benign solvents, and lower-temperature processing routes. Figure 2 shows the comparative evolution of green versus conventional synthesis routes for AWH materials.

A crucial trade-off between sustainability and operational robustness is highlighted by the fact that, although bio-derived nanomaterials can lessen the environmental effects of the synthesis stage, their long-term durability and scalability under repeated AWH cycling are still less established than those of conventionally synthesized inorganic sorbents. For AWH-relevant compounds, Table 3 offer illustrative examples from experimental and review investigations that compare green and conventional synthesis methodologies. While Table 3b emphasizes qualitative factors pertaining to energy requirements, by-product toxicity, and relative carbon footprint, Table 3a summarizes synthesis pathways and processing conditions. It is important to note that the presented figures are indicative of comparative trends rather than absolute life-cycle standards because they reflect different techniques, scales, and system boundaries.

In comparison to traditional solvothermal or chemical approaches, green synthesis routes often show relative reductions in processing energy and solvent-related impacts on the scale of tens of percent throughout the assessed research, especially for oxide- and silica-based compounds. For instance, high-boiling organic solvents and high reaction temperatures are usually avoided in the aqueous or bio-assisted synthesis of ZnO, FeO_3_, and SiO_2_-based materials, which lowers the environmental load associated with solvents and direct energy input. Similar to this, water-based synthesis of MOF-801 and MOF-303 reduces reported embodied emissions at similar laboratory-scale circumstances by doing away with DMF and related solvent recovery procedures.

However, pore design, surface chemistry, and post-synthesis treatment continue to have a significant impact on the functional performance of AWH materials made by environmentally friendly methods, including water absorption capacity, regeneration temperature, and cycling stability. This emphasizes how crucial it is to assess green synthesis techniques within a more comprehensive mechanistic and life-cycle framework because improvements in sustainability at the synthesis stage would not always result in better system-level performance.

The comparative trends displayed in Table 3 indicate that green synthesis techniques offer a workable, if conditional, way to reduce the environmental effect of creating AWH products. By lowering energy intensity and minimizing solvent-related risks during synthesis, green nanotechnology enables more sustainable deployment of AWH systems while maintaining adsorption capacity, regeneration efficiency, and long-term durability under practical operating settings.

## 4. Application of Green Nanotechnology in Atmospheric Water Harvesting

Green nanotechnology has made it possible to create AWH materials that balance water uptake capacity, regeneration energy demand, and scalability. Metal oxides, graphene-based composites, metal–organic frameworks (MOFs), and bio-derived aerogels are some of the most researched material classes for sorption-based and condensation-assisted AWH systems. Through nanoscale alteration of surface chemistry, pore architecture, and thermal transport capabilities, these materials have been evaluated in laboratory-scale studies and particular pilot or field demonstrations under diverse climatic conditions [15,28,68,70,72,76,77]. Although environmental factors and system configuration still have a big influence on outcomes, reported results show that material design is crucial in influencing water yield, regeneration feasibility, and operational robustness.

### 4.1. Nanomaterials for Green AWH Systems

#### 4.1.1. Metal Oxides and Inorganic Nanomaterials

AWH benefits from metal oxide nanoparticles in two primary ways. In dew and fog collecting devices, they first enhance condensation on surfaces. Second, because of their high-surface-area and porous designs, they aid in vapor absorption. Due to its high surface energy and ability to produce intricate micro- and nanoscale patterns, zinc oxide (ZnO) nanostructures are frequently utilized in condensation-based AWH. Flower-like ZnO arrays make the surface extremely hydrophilic when grown on textiles or mesh, facilitating droplet formation, merging, and sliding off. As long as the humidity and radiative cooling conditions are appropriate, field tests have demonstrated that these coatings can collect water at night without requiring additional energy. This suggests that ZnO coatings could be an inexpensive, passive method of collecting dew [71].

In order to modify surface wettability and add photocatalytic or photothermal capabilities, titanium dioxide (TiO_2_) nanostructures are frequently included in fog and dew-harvesting meshes. When exposed to UV or solar radiation, the anatase phase of TiO_2_ lowers the nucleation barrier for droplet formation and permits periodic surface heating that decreases surface fouling and flooding. Although these mechanisms help improve long-term operational stability in high-humidity or fog-dominated situations, environmental exposure and radiation intensity still have an impact on overall performance [72,78].

An advanced inorganic sorbent for vapor-phase water capture in AWH is mesoporous silica. Because of its enormous surface area and interconnected pore networks, hollow core–shell mesoporous silica capsules absorb more water than conventional silica gel. Adsorption capacities beyond 0.32 g H_2_O/g at 25 °C and 40% relative humidity are a sign of effective performance in moderate humidity conditions. Engineered silica is commonly employed as a reference sorbent in the design of adsorption-based water-harvesting systems due to the relative ease of scaling up the sorbent preparation and the reduced regeneration temperatures required. However, the long-term stability of the sorbent and mechanical strength are of critical concern [15].

#### 4.1.2. Graphene Oxide-Based Sorbents

Graphene oxide (GO) and reduced graphene oxide (rGO) are considered to be a paradigm shift in the development of hybrid AWH sorbents. Although graphene is known to be extremely hydrophobic in nature, the chemical modification of graphite leads to the formation of hydroxyl, epoxy, and carboxyl groups on the graphene surface. These groups make GO extremely hydrophilic, creating a massive two-dimensional platform for water capture [79]. In AWH systems, GO has several functionalities, such as heat conductivity, photothermal absorption, and vapor transmission. Recent studies using molecular dynamics and density functional theory have identified that the water affinity of GO can be exponentially enhanced by accurately controlling the intercalation of atoms [79]. By incorporating alkaline earth metal cations into the GO matrix, the interface between the solid and liquid phases has been significantly polarized. In Ca-GOA, the combined effect of the synergistic interaction between the calcium cation and the adjacent epoxide functional group has significantly enhanced the hydrogen bond enthalpies of the adjacent water molecules. In this case, the Ca-GOA surface has the capability to capture times more water molecules compared to the free-standing GO structure in the absence of any dopant [79].

In MOF-GO hybrid materials consisting of a laminated structure of MOFs and GOs, the presence of GOs has a positive effect on the intercalated structure, thus facilitating heat transfer and vapor transmission. The equilibrium water uptake of these materials remains unchanged. The presence of GOs in these materials has a positive effect on heat transfer and vapor transmission. The adsorption half-time of these materials, such as MIL-101(Cr)/GO, is 35% lower than that of pristine MOFs when humidity is low. The water uptake of these materials remains unchanged at 0.30 g H_2_O/g of material [24,80].

In salt-based sorbent materials, graphene aerogel materials have been used to form a strong structure. For instance, LiCl-loaded holey graphene aerogel fibers have a water uptake capacity of over 4 g of water per gram of material. These materials can be quickly activated under solar irradiance. The materials have a high water uptake capacity. The presence of a graphene structure allows for even heat transfer under solar irradiance. The regeneration of these materials can be sped up under solar irradiance. These materials have a high water uptake capacity, but they can only function well under humid conditions. The stability of salt in these materials must be carefully monitored to ensure that salt leakage does not occur after regeneration [63]. The confinement of LiCl in a vertically aligned reduced graphene oxide and sodium alginate matrix (LiCl@rGO-SA) provides a robust and leak-free sorbent structure [81]. The vertical alignment of the rGO sheets provides a direct and untortuous path for the diffusion of water vapor, thus enhancing the rate of mass transport by orders of magnitude [81].

Crucially, rGO acts as an elite broadband photothermal converter [82]. Within the desorption phase, the rGO sheets absorb the incoming light and directly focus this thermal energy to the solid–water interface within the pores. This ensures that no heat is lost to the surrounding material or air, a problem with conventional sensible heating methods [83]. As a result, the LiCl@rGO-SA composite is able to rapidly attain regeneration temperatures of 70–90 °C under standard 1-sun illumination and complete a full desorption cycle in just minutes [83]. This ultra-fast cycling capacity of the composite enables it to carry out up to eight cycles of capture–release daily, resulting in a staggering daily water productivity of 2.12 L∙kg^−1^∙day^−1^ directly from arid air without any external electrical energy supply [82].

#### 4.1.3. Metal–Organic Frameworks

Metal–organic frameworks (MOFs) have emerged as the most investigated materials for sorption-based atmospheric water harvesting. They display distinct, stepwise water adsorption isotherms, crystallinity, and tunable pore chemistry. The advantage of using MOFs in dry and near-dry conditions is that their RH trigger for fast water uptake can be finely tuned by selecting different metal nodes, organic linkers, and pore configurations [84].

The benchmark materials in this class are the zirconium-based MOF-801 and the aluminum-based MOF-303. The adsorption mechanism within these frameworks relies on highly specific pore-filling sequences. In the case of MOF-303, the initial water molecule enters the pore and strongly adsorbs to the polar N(H) group of the pyrazole linker through three distinct hydrogen bonds [14]. This initial water molecule then acts as an anchor for subsequent water molecules, which adsorb to it rather than the framework and rapidly form a highly ordered water cluster to trigger capillary condensation and fill the pore. This results in a very distinct S-shaped isotherm, indicating that the material adsorbs virtually zero water at 10% RH, but immediately attains maximum capacity at 20% RH, and desorbs all of it with even a small increase in temperature [85]. Field testing has demonstrated the practical viability of these materials. In the hyper-arid environment of the Mojave desert, a passively cooled device employing MOF-303 was shown to provide 0.7 L∙kg^−1^∙day^−1^ while operating within a range of 10–20% RH day–night cycles. In controlled indoor testing at 32% RH, optimized MOF-303 configurations have been shown to attain water production rates as high as 1.3 L∙kg^−1^∙day^−1^ [85]. On other hand, MOF-801 (Zr-fumarate) is recognized among benchmark systems for its reliable water-harvesting performance in low relative humidity conditions when mass- and heat-transfer restrictions are appropriately addressed. Its suitability for low-grade or solar-driven regeneration is demonstrated by reports of daily water yields of up to 2.8 L·kg^−1^·day^−1^ at roughly 20% relative humidity using regeneration temperatures below 70 °C [30]. These features are caused by the hydrophilic zirconium oxide clusters and pore sizes that encourage capillary condensation at low vapor pressures.

Recent studies indicate that temperature control and framework design are crucial performance factors in metal–organic framework (MOF)-based atmospheric water harvesting (AWH), in addition to inherent adsorption behavior. By actively regulating the heat fluxes associated with adsorption and desorption inside the sorbent bed, for example, thermoelectrically controlled MOF monoliths have been shown to boost water productivity significantly. A sandwich-structured MIL-101(Cr) monolith coupled with a thermoelectric cooler generated continuous water production of approximately 3.7 L·kg^−1^·day^−1^ at 30% relative humidity and 25 °C, illustrating how framework chemistry and device-level heat regulation can address kinetic and thermal limitations in arid environments [86].

Complementary advances in framework chemistry and topology further expand the MOF design field relevant to AWH. Humidity-responsive adsorption behavior is indirectly related to pore accessibility, hydrogen-bonding networks, and framework stability, all of which are highly impacted by cis/trans octahedral coordination and linker-induced topological alterations, according to recent structural investigations [87]. In addition to gas separation and catalysis, atmospheric water harvesting is an emerging application area, according to a detailed analysis of the top-performing MOFs from 2014 to 2024. It also highlights the increasing significance of mixed-linker approaches and data-driven, AI-assisted material discovery frameworks in maximizing sorption performance [88].

Practical issues arise regarding form, heat release, and cost as MOF sorbents transition from the powdered state to real-world applications in AWH systems. Thus, the focus is on structured MOF composites, binder-made monoliths, and hybrid materials that improve heat release capabilities without sacrificing adsorption performance. MOFs serve as a demo in the larger AWH world, illustrating the relationship between system renewal capabilities and life-cycle sustainability in relation to the nanoscale adsorption phenomenon.

#### 4.1.4. Covalent Organic Frameworks (COFs)

Addressing the stability limitations inherent to coordinate-bonded networks, Covalent Organic Frameworks (COFs) represent the absolute bleeding-edge of reticular AWH chemistry [89]. COFs are purely organic, porous crystalline polymers constructed entirely from light elements (C, H, N, O, B) linked via exceptionally strong, irreversible covalent bonds (e.g., imine, hydrazone, ketoenamine). This purely covalent architecture grants COFs near-invulnerability to hydrolytic degradation, maintaining structural integrity across highly acidic, basic, and continuously boiling water environments that would quickly dissolve many MOFs. Furthermore, lacking heavy metal nodes, COFs possess intrinsically lower skeletal densities, maximizing the volume-to-mass ratio for active vapor capture [89].

The thermodynamic profiles of tailored COFs are highly favorable for AWH. By strategically integrating hydrophilic functional moieties into the organic backbone, researchers can engineer COFs to exhibit ideal S-shaped isotherms with near-zero hysteresis. For instance, COF-432 exhibits a sharp pore-filling step at relative humidities below 40% and demonstrates total retention of its working capacity even after 300 rigorous adsorption–desorption cycles [90].

Recent advancements in ketoenamine-linked frameworks have pushed kinetic boundaries further. The COF-ok material has demonstrated a robust working capacity of 0.4 g∙g^−1^ at just 30% RH [89]. Because the desorption enthalpies are highly optimized, COF-ok can undergo complete regeneration under mild solar heating (45 °C), allowing for rapid, continuous daytime cycling. Under outdoor testing conditions using solely passive solar energy, continuous-cycle devices employing COF-ok have reported total daily yields of 161 g·kg^−1^·day^−1^ [89]. While their gravimetric capacities at high humidity values may currently trail swelling hydrogels, their absolute durability and ultra-low regeneration energy thresholds firmly establish COFs as premier candidates for long-term deployment in hostile arid zones.

#### 4.1.5. Bio-Derived and Photothermal Aerogels

Bio-derived aerogels composed of chitosan, cellulose nanofibers, alginate, and other biopolymers have been recognized as potential AWH media. They provide the benefits of low-temperature processing, bio-renewability, and the ability to retain hygroscopic salts or active nanoparticles while retaining mechanical properties. Composites of bio-derived aerogels and other materials such as graphene, carbon nanotubes, and carbonized biomass have been used for solar-driven water regeneration.

Aerogels that have channel-like structures and vertical orientations have been reported to show enhanced adsorption kinetics and regeneration efficiency. Studies have demonstrated the water adsorption capacity of bio-derived aerogels in the range of 0.7 g H_2_O/g of material at low relative humidity and 4 g H_2_O/g of material at high relative humidity [91]. However, the water adsorption capacity of the aerogels depends on the salt content and ambient conditions [76,92]. Studies on the long-term stability of the aerogels have been conducted. However, the aerogels have demonstrated the ability to perform effectively in multiple adsorption and regeneration cycles.

Bio-derived and photothermal aerogels have been recognized as high-performance AWH materials. They have the potential for water harvesting and regeneration while providing the benefits of biodegradable properties, hierarchical structures, and low-energy regeneration. However, the effectiveness of the aerogels in AWH has highlighted the necessity of system-level optimization.

The material classes covered in this section demonstrate that no single green nanomaterial provides consistently excellent performance for atmospheric water harvesting under all operating situations. In systems based on condensation and fog, where performance is governed by surface wettability, durability, and passive operation, metal oxides and inorganic nanostructures are very advantageous. Despite compromises in mechanical robustness and long-term stability, mesoporous silica and bio-derived aerogels perform exceptionally well in moderate-to-high humidity regimes by encouraging capillary condensation and permitting low-temperature or solar-driven regeneration. However, because of their steep, humidity-triggered adsorption isotherms, metal–organic frameworks are particularly well adapted for low relative humidity settings. However, their actual implementation depends on regeneration design, synthesis scalability, and shaping approach. These variations highlight the importance of matching material qualities to specific environmental conditions, regeneration processes, and life-cycle constraints. They also provide the foundation of the subsequent comparative analysis.

### 4.2. Nano-Engineering Strategies for Efficiency Enhancement

This section will discuss the ways in which graphene-based devices perform in the context of the adsorption-water harvesting system, including the ways in which the materials can be efficiently restored and how the transport of the vapor can be maximized. Apart from the materials’ chemical properties, the role of nano-engineering techniques in improving the efficiency of these materials is significant. These techniques primarily enhance adsorption and desorption rates, ensure system stability over long periods, reduce the energy required for restoration, and increase the overall sorption capacity at equilibrium [4,6,11,29]. Among these techniques, the design of surface wetting, the creation of desiccant nanocomposites, and the use of photothermal restoration are the most significant.

#### 4.2.1. Surface Wettability Engineering

In AWH systems that use condensation and fog, surface wettability controls the kinetics of droplet nucleation, coalescence, and removal [19,23]. While superhydrophilic surfaces hasten the nucleation and dispersion of condensed water, superhydrophobic areas encourage droplet shedding and reduce surface flooding. Recent biphilic and patterned designs deliberately combine these features to produce capillary pressure gradients that direct water flow and enhance collection efficiency [72].

A copper-fabric fog collector with alternating TiO_2_–silica superhydrophilic pads and fluorosilane-treated superhydrophobic channels is used to illustrate this technique. Compared to uniformly hydrophilic meshes, this biphilic design increased water collection rates by around four times under controlled fog-tunnel conditions (25 °C, 75–85% RH). The apparatus continuously generated water flows of 2.5–3 L m^−2^ day^−1^ over a six-month field deployment along the Namibian coast [72]. Over time, however, wettability-gradient collectors demonstrated decreased droplet mobility and increased contact angles as a result of gradual UV-induced ageing, which resulted in a significant durability trade-off [72].

#### 4.2.2. Desiccant Nanocomposite Architectures

Instead of optimizing intrinsic sorption capacity, nano-engineering techniques for adsorption-based AWH systems mainly concentrate on reducing diffusion resistance, enhancing mechanical integrity, and permitting useful form factors [4,6,29]. Even though powdered sorbents, like MOFs, frequently show high equilibrium uptake, repeated cycling can cause delayed vapor transport, particle attrition, and ineffective heat dissipation [17,25,26,28]. By developing designed diffusion channels and mechanically stabilized designs, embedding these sorbents within structured polymeric or biopolymeric matrices provides a solution to overcome these constraints [70].

This method is demonstrated by hierarchically porous monoliths created by in situ crystallization of MOF-801 within chitosan/poly(vinyl alcohol) (PVA) hydrogels. While the polymer binder reduces dusting and makes cartridge-type deployment easier, vertically oriented micro- and mesoporous channels shorten vapor diffusion pathways in comparison to loose powders, allowing for faster cycling and better handling [70]. Additional evidence of improved mechanical robustness under varying ambient humidity conditions comes from rooftop testing and prolonged cycling tests [70]. Ongoing efforts to reduce binder content while maintaining channelized transport and structural integrity are motivated by the additional factors that polymer matrices introduce, such as dehydration under severe temperatures and binder-related costs [4,70].

#### 4.2.3. Photothermal-Enhanced Regeneration

Photothermal regeneration in nano-enabled AWH systems uses a single mechanism in which nanostructured absorbers transform incident sunlight into localized interfacial heat. This approach reduces bulk thermal losses, accelerates desorption kinetics, and removes adsorption enthalpy barriers. Photothermal enhancement enables electricity-free regeneration by converting solar energy into localized heat at the sorbent interface. Carbon-based nanomaterials, such as carbon nanotubes (CNTs) and graphene derivatives, are widely employed as photothermal converters due to their efficient heat transmission and broadband light absorption. They have also been demonstrated to speed up desorption kinetics in sorption-based AWH systems [24,63]. Regeneration techniques that preserve cycling stability and very small temperature changes are advantageous for MOF-based systems [25,26,28].

A sodium alginate/CNT/MgCl_2_ aerogel is capable of absorbing solar light to generate heat, increasing local temperature. This allows for multiple adsorption–desorption cycles without any external power sources [76]. The potential of this method for autonomous harvesting of atmospheric water at different humidity levels is significant, as it is capable of delivering substantial quantities of water at high humidity, as well as regeneration under solar power [76]. However, there are challenges to be overcome, including those of cost and energy consumption in CNT synthesis, as well as those of deliquescing salt movement, which could compromise long-term structural integrity. The focus of current research is to find ways to retain the salt within cross-linked materials, as well as using alternative, environmentally friendly photothermal materials, such as biomass-derived carbons [76].

#### 4.2.4. Integrated Perspective

Instead of focusing only on equilibrium capacity, nano-engineering solutions concurrently address major performance bottlenecks in AWH systems, such as poor sorption kinetics, inefficient heat management, and deterioration with repeated cycling [4,6,11,29]. Wettability gradients maximize liquid transport in condensation and fog harvesting [19,23,72], structured nanocomposites accelerate vapor diffusion and enable deployable form factors [70], and photothermal interfaces isolate regeneration from grid electricity [24,63,76]. In addition to making it clear where durability, material cost, and life-cycle effect need to be carefully managed to ensure scalable and sustainable deployment, the convergence of these techniques enables the development of small, low-energy AWH systems that can function in a variety of climatic conditions [4,6,29].

In AWH systems, ordered and disordered pore designs impose different restrictions on heat dissipation, vapor mobility, and cycle stability. Examples of disordered designs that typically have a large intrinsic surface area and advantageous equilibrium absorption include granular composites, powder beds, and random aerogels. However, the complex and long diffusion routes of their winding pore networks may limit adsorption kinetics at low relative humidity and hinder effective heat removal during regeneration. These restrictions frequently show as slow absorption rates, temperature gradients within the sorbent substrate, and partial desorption under mild regeneration conditions, especially after repeated cycling.

On the other hand, directed freeze-cast aerogels, honeycomb monoliths, lattice scaffolds, and vertically aligned channels are examples of organized structures that decrease effective diffusion length and encourage more consistent vapor access to active regions. Even when equilibrium sorption capacity is similar to that of disordered equivalents, ordered structures accelerate adsorption–desorption cycling by improving mass-transfer kinetics and mitigating internal concentration gradients through continuous and preferred transport channels. Furthermore, more uniform regeneration under solar or low-grade thermal input is made possible by aligned pore networks’ enhanced thermal conductivity and heat dissipation.

Crucially, kinetic and thermal effects—rather than higher sorption capacity—are the main sources of ordered designs’ performance benefits. Because they maintain greater cycling frequencies and more thorough regeneration, structured monoliths and lattices frequently outperform disordered powders in terms of daily water productivity under equivalent humidity circumstances. However, there are trade-offs associated with these advantages, such as difficulties in large-scale manufacture, possible mechanical anisotropy, and higher fabrication complexity.

The literature still lacks direct, side-by-side experimental comparisons between ordered and disordered pore designs carried out with the same airflow, temperature, humidity, and cycle protocols. Therefore, rather than being normalized quantitative standards, observed performance disparities should be seen as mechanistically informed trends. In order to thoroughly evaluate the actual contribution of pore ordering to AWH performance, this gap emphasizes the necessity for standardized testing frameworks that separate intrinsic sorbent chemistry from architectural impacts.

### 4.3. Representative Studies and Performance Benchmarks

The real-world performance of AWH materials must be evaluated under real-world environmental conditions, including fluctuating humidity, daily temperature variations, and multiple cycles of adsorption–desorption. Recently, there has been a move towards outdoor testing, pilot-scale testing, and cycling experiments that test the performance of the materials beyond the equilibrium capacity.

The daily water production capacity of nano-enhanced AWH systems differs by over tenfold. The wide range in the performance of AWHs results from the inherent properties of the materials, the operation mode, climate, regeneration strategy, and system design. To give an example, passive MOF-based AWHs in arid environments such as the Mojave Desert are reported to produce approximately 0.6–0.8 L kg^−1^ day^−1^ at relative humidity below 25% [28]. On the other hand, the performance of fan-assisted AWHs using MOF materials reaches approximately 3–5 L kg^−1^ day^−1^ at the same environmental conditions. The higher performance comes at the expense of increased specific energy consumption (1.7–5.3 kWh L^−1^) [93].

By addressing kinetic and thermal constraints at the material and structural levels, nano-engineered composite structures provide an intermediate solution. Faster adsorption–desorption cycles under low-grade solar input or moderate heating are made possible by structured MOF-polymer monoliths and channeled aerogel sorbents, which lower diffusion resistance and enhance heat dissipation [70,92]. With reported daily water yields approaching 10 L kg^−1^ day^−1^ under humid subtropical conditions, photothermal aerogels incorporating carbon nanostructures or bio-derived absorbers show high water uptake across wide RH ranges (approximately 20–95% RH) and support autonomous solar-driven regeneration [94].

A comparable class of nano-enabled AWH systems that prioritize passive regeneration and wide-humidity operability over maximum equilibrium capacity are salt-confined polymeric or aerogel-based hygroscopic lattices. Under low-to-moderate relative humidity conditions, outdoor or quasi-field tests of such structured hygroscopic composites reveal typical water productivities of roughly 0.6–0.7 L kg^−1^ day^−1^, with adsorption–desorption cycle durations in the range of tens of minutes [77]. Although low regeneration temperatures and electricity-free operation are advantages of these systems, long-term performance is still dependent on mechanical stability, environmental exposure, and salt containment technique.

Systems based on condensation and fog that are improved by nanostructured surfaces function within a specific performance regime. Water flows of about 2–4 L m^−2^ day^−1^ are achieved by biphilic TiO_2_/silica meshes placed in fog-rich coastal areas without electrical input, demonstrating their feasibility for deployment that is energy-independent but geographically restricted [95]. Nevertheless, these systems continue to be extremely site-specific and susceptible to surface ageing, fog density, and wind.

There is still a lot of uncertainty about cross-study comparison. Direct quantitative comparison between platforms is complicated by the fact that reported water yields depend on various regeneration temperatures, cycle durations, humidity calibration techniques, and normalization protocols [4,29]. Furthermore, statistical variation, multi-seasonal field datasets, and long-term deterioration measures are reported in very few studies. Although mechanisms including salt migration, mechanical attrition, and UV-induced wettability drift are commonly recognized, they are rarely measured over long working times [70,76,77]. When comparing structured and disordered AWH systems, condition-matched benchmarking is necessary because the apparent performance advantages of ordered architectures noted in field and outdoor studies are primarily due to enhanced vapor transport and regeneration kinetics rather than inherent differences in equilibrium sorption capacity.

**Figure 3:**
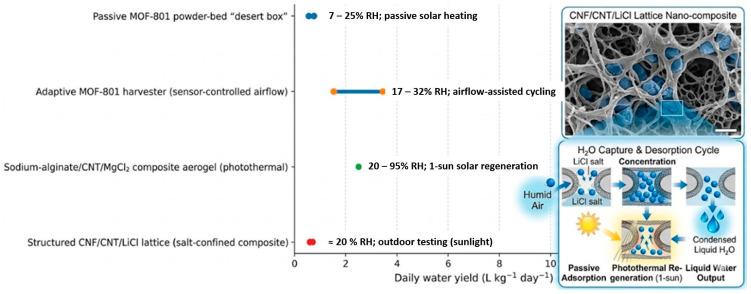
Reported ranges of daily water production for representative nano-enabled AWH systems evaluated under field or extended outdoor conditions [24,56,64,65]. Values reflect study-specific operating conditions, system architectures, and regeneration strategies rather than enabling direct quantitative comparison. Note: Ranges represent reported or representative daily water yields measured under study-specific field or extended outdoor conditions; values are not normalized across systems.

Representative nano-enabled AWH systems that have advanced beyond brief laboratory testing, such as field deployments and prolonged cycling investigations, are compiled in Table 4. The modes of operation, regeneration techniques, and study-specific considerations all contribute to the set of performance numbers we see because they come directly from the original studies. Figure 3 helps put these data in context and narrows it down to what constitutes acceptable ranges instead of ranking systems by displaying the ranges of daily water yield for specific setups that have been tested under outdoor or field-like conditions. Since many studies use small cycling data sets or test a single site in a field test, repeated testing under standardized conditions is not always an option. Therefore, we should consider the collected data to be indicative of a set of performance envelopes rather than a set of data that has converted to a specific value.

### 4.4. Design Implications from Field Benchmarks

Together, Table 4 and Figure 3 show certain recurrent trends that are pertinent to AWH system design in the future. First, vapor-transport kinetics and temperature regulation are at least as important to actual water yield as equilibrium sorption capacity. Architectures that enable rapid mass and heat transport can occasionally outperform slow-adsorbing powder beds while having lower intrinsic absorption. Second, water production is often power-constrained: totally passive or solar-powered systems minimize electrical input at the cost of decreased throughput and heightened susceptibility to environmental degradation. Fan-assisted systems, on the other hand, produce more but use a lot more energy. Lastly, system design is becoming more constrained by sustainability issues. Green synthesis methods and biopolymer scaffolds can lower life-cycle emissions; these benefits could be countered by high-impact additions or unstable salts. Instead of focusing on discrete increases in sorbent capacity, these findings highlight the necessity of coordinated optimization across material selection, system architecture, and life-cycle performance.

## 5. Challenges, Durability, and Future Directions for Green Nano-Based Atmospheric Water Harvesting

When compared to traditional methods, recent laboratory and pilot-scale studies have demonstrated that nano-engineered materials can greatly increase AWH efficacy. Photothermal composite aerogels have shown high daily yields during outdoor testing in humid situations [76,92]. At the same time, vertically structured MOF-based architectures have decreased sorption-cycle times and improved practical productivity under controlled conditions [25,28,34]. When compared to equally wetted controls, biphilic TiO_2_/silica meshes have demonstrated approximately twofold increases in fog-collection efficiency [71,72]. Issues with material lifetime, manufacturability, energy autonomy, and life-cycle sustainability continue to impede the transition from proof-of-concept devices to dependable, scalable infrastructure [4,6,11,29].

### 5.1. Material Durability, Hygienic Stability, and Aging Mechanisms

Maintaining adsorption capacity, surface wettability, and hygienic integrity in the face of frequent humidity–temperature cycling and extended outside exposure is necessary for sustained AWH functioning [4,6,29]. Long-term durability beyond initial sorption performance increasingly determines the viability of nano-enabled AWH systems, particularly in decentralized or off-grid deployments where maintenance opportunities are limited.

Through surface-mediated inhibition mechanisms, such as the production of reactive oxygen species, photocatalytic activity, or disruption of microbial cell membranes, green-synthesized or bio-assisted metal and metal-oxide nanomaterials (such as ZnO, TiO_2_, and Fe-based particles) are often reported to exhibit antimicrobial or anti-biofouling behavior [58,59,62,63]. These characteristics could reduce the growth of microorganisms on sorbent surfaces and wetted collectors. In water-treatment applications, bio-derived and biogenic nanomaterials have also been investigated as less harmful substitutes, providing sufficient functional stability together with decreased solvent toxicity and embodied energy [64]. Nevertheless, antibacterial activity is not universal and is highly dependent on long-term surface chemical evolution, particle dispersion, surface accessibility, and lighting conditions.

Microbial fouling and biofilm formation have been explicitly reported for hydrophilic polymeric and aerogel-based materials operated under humid or cyclic wet–dry conditions. For instance, long-term exposure to ambient humidity has caused microbial growth and organic fouling in cellulose- and alginate-based aerogels used in water adsorption, dehumidification, and filtration systems, resulting in pore blockage and decreased mass-transfer kinetics [92,96,97]. Similar biofilm formation has been observed on highly hydrophilic polymer-supported sorbents and membranes in air–water interface and water-treatment applications, where sustained moisture availability promotes microbial colonization, surface aging, and loss of functional efficiency [98,99]. These observations indicate that high hygroscopicity alone does not guarantee hygienic stability in long-term AWH operation. Antimicrobial oxides (e.g., ZnO, TiO_2_), periodic thermal or solar-assisted regeneration to inhibit microbial growth, and surface functionalization techniques intended to decrease bio-adhesion while maintaining water uptake and cycling performance are examples of reported mitigation strategies [58,59,62,63,100].

As long as heat and mass transfer are properly controlled and contaminant exposure is kept to a minimum, a number of MOF materials have shown stable cyclic operation for sorption-based AWH systems throughout hundreds of adsorption–desorption cycles in laboratory and field-oriented research [25,26,28,34]. However, outdoor deployment creates new ageing routes that are still not well described at the system level. These include (i) UV-induced surface ageing and wettability drift of condensation and fog-collection coatings [71,72], and (ii) migration (also known as “creep”) and partial loss of deliquescent salts (such as LiCl and MgCl_2_) within composite sorbents, which can cause mechanical fatigue, corrosion, or pore blockage during repeated cycling [76,77,92].

Photostable surface treatments, ligand and surface-chemistry optimization, encapsulation or confinement of hygroscopic phases, and structural designs—like aligned channels, lattices, or scaffolded architectures—that improve mechanical stability and active component retention while enhancing vapor transport are examples of mitigation strategies documented in the literature [71,72,76,77,92]. These results show that durability, hygienic stability, and ageing behavior are co-equal design constraints with adsorption capacity and regeneration efficiency when transferring green nanomaterials from laboratory demonstrations to deployable AWH systems.

### 5.2. Environmental, Manufacturing, and Scale-Up Constraints

Although green synthesis methodologies generally reduce solvent toxicity and processing energy when compared to conventional methods, scale-up presents practical challenges with repeatability, cost, and supply-chain consistency [11,45,46,65,73,101]. Significant geographical and seasonal fluctuations in plant-based extracts and agricultural-waste feedstocks complicate batch-to-batch control during nanomaterial synthesis and surface functionalization [46,47,50,65]. The cost of materials and manufacturing complexity is still higher than those of well-known desiccants like silica gel or zeolites, even though aqueous and lower-temperature synthesis methods for metal–organic frameworks (MOFs) are improving [11,28,29,30,31,34]. This is especially true when high-purity linkers, shaping aids, and corrosion-resistant housings are required.

At the device level, salt leaching, long-term structural deterioration, and nanoparticle aggregation can reduce performance repeatability and raise maintenance requirements during cyclic operation [29,76,77,96]. Crucially, sustainability needs to be assessed at the system level as opposed to just the sorbent level. In order to evaluate embodied energy, carbon emissions, and end-of-life impacts, including the possible release or environmental fate of nano-additives, and to support responsible deployment choices, life-cycle assessment (LCA) carried out under ISO 14040/14044 frameworks is becoming more and more advised [73]. The requirement for clear performance, durability, and risk documentation is being reinforced by the growth of regulatory engagement through documented demonstrations and material registries for certain classes of nano-enabled environmental solutions [68,69].

From a life-cycle viewpoint, documented cradle-to-gate embodied energy values for conventional sorbents such as zeolites and silica gel typically range from 5 to 15 MJ kg^−1^. Conversely, solvothermally synthesized metal–organic frameworks (MOFs) can exceed 50 to 100 MJ kg^−1^, depending on the synthesis temperature and solvent recovery efficiency. When compared to DMF-based solvothermal approaches, recent water-based or bio-assisted MOF synthesis strategies exhibit embodied energy reductions of roughly 30–60%, underscoring the importance of synthesis route selection in determining overall sustainability [102]. However, if photothermal additives, deliquescent salts, or frequent sorbent replacement account for a significant portion of the material mass or operational lifetime, these advantages may be negated at the system level. This emphasizes the necessity of life-cycle assessment (LCA) at the system level as opposed to material-only comparisons.

The integrity of a life-cycle approach is implicitly dependent on a transition from milligram-level laboratory synthesis to ton-level industrial production. Without economic viability, even a theoretically perfect nanomaterial from a thermodynamic perspective remains an academic curiosity. Historically, the scaling up of the commercialization of MOFs has been hindered by the use of the solvothermal technique, which involved the use of massive volumes of toxic and high-boiling solvents like DMF. The energetic costs involved in the recovery of these solvents, in addition to environmental regulations for disposal, have made the costs of producing baseline MOFs exceed $500 kg^−1^ [103]. However, the adoption of green chemistry has transformed the scaling up of the commercialization of MOFs.

Recent advances have shown the capability for continuous and kilogram-scale fabrication of benchmark water-harvesting MOFs such as MOF-303, MIL-160, and CAU-10 by purely aqueous and ambient-pressure routes. These “green” synthesis routes, performed in 200-L pilot-scale batch reactors, exploit the use of inexpensive and ubiquitous starting materials such as aluminum sulfate and sodium hydroxide to deliver outstanding space-time yields of between 238 and 305 kg∙day^−1^∙m^−3^, with reaction yields consistently exceeding 90% [103]. The results of the techno-economic analysis (TEA) of the “green” routes and alternatives such as liquid-assisted grinding indicate that the cost of the industrial-scale synthesis of robust MOFs will be driven down to a mere $13–$30 per kilogram, thereby directly targeting the key cost threshold of ≤$10 per kilogram that is necessary for the commoditization of such materials for the mass market [18].

The mass production of Graphene Oxide (GO) poses unique, yet conquerable, economic hurdles. For instance, conventional production of GO by means of the Hummers’ technique is known to produce extremely toxic gaseous byproducts and demands huge amounts of water for purification, posing considerable environmental hurdles [43]. Nevertheless, alternative “green” routes to GO production, such as those involving citric acid reduction and crossflow filtration, have overcome such hurdles to make GO production commercially viable. Although high-purity graphite feedstocks account for 50–60% of the costs in GO production plants, the tremendous, interdisciplinary need for GO ensures that production volumes remain high enough to sustain gross profit margins of 45–60%.

Currently, Covalent Organic Frameworks (COFs) possess the steepest economic hurdles to overcome [104]. Although their elemental constituents are often very inexpensive, to obtain high levels of crystallinity, which are needed to obtain optimal AWH performance, reaction times are often very long, organic catalysts are rare and expensive, and very strict atmosphere control is required [104]. However, with rapid breakthroughs in mechanochemical and continuous-flow flux synthesis routes, it is clear that COF costs will soon fall with further progression of the learning curve in their production [105].

At the systemic deployment level, the economic viability of AWH is highly dependent upon the type of energy source employed in the desorption cycle. In fact, rigorous TEA revealed that the Levelized Cost of Water (LCOW) for active and grid-connected AWH systems varies between $6.50 and $11.00 per m^3^, contingent upon environmental humidity and prevailing electricity rates [31]. However, when these systems are integrated with depreciated existing solar photovoltaic systems or photothermal interfaces, the major operating cost for AWH systems completely disappears [31]. In fact, under optimized solar configurations, the LCOW for AWH systems drops to as low as $0.09 per L in arid environments and as low as $0.02 per L in tropical environments [31]. At these levels, AWH systems emerge as a highly competitive and decentralized pillar in the global water infrastructure.

End-of-life (EoL) behavior is another barrier to the general application of nano-enabled AWH systems beyond the production and operating phases. Because of their exceptional thermal and chemical robustness, inorganic sorbents, such as metal oxides and some metal–organic frameworks (MOFs), may often be reactivated, recycled, or reused through heat treatment. On the other hand, bio-derived polymers, hydrogels, and aerogels exhibit high exposure to oxidation, hydrolysis, or microbial degradation. Therefore, this may reduce their environmental persistence as well as compromise their long-term structural integrity. In addition, there are other challenges with the component separation, salt leakage, and environmental destiny originating from hybrid materials that comprise salts, carbon nanostructures, or polymer binders [76,77,92]. There are currently few reported recycling or disposal methods for AWH sorbents, and end-of-life consequences are rarely formally evaluated. Therefore, the overall sustainability of the AWH methods can be improved, along with reducing the environmental impacts by integrating recyclability, benign degradation, or controlled material recovery into sorbent and system design.

### 5.3. Research Priorities and Strategic Roadmap

An integration of materials science, system engineering, and chemical engineering, along with standardized validation, will be performed to advance the development of green nanomaterials-based AWH systems. More specifically, the hybrid sorbents combine the high uptake at low relative humidity with fast vapor transport and low-temperature regeneration [25,26,28,34,76,92]. In addition, structural architectures control heat and mass transfer through aligned channels to enhance kinetics without compromising durability [25,76,77,92]. Finally, durability-by-design strategies address UV ageing, salt retention, corrosion, and biofouling in realistic field conditions [71,72,76,77,92].

Future research should emphasize standardized reporting of operating conditions, such as relative humidity, temperature, cycle duration, regeneration mode, and water quality, as well as clear discussion of uncertainty and degradation behavior, given the increasing variety of reported AWH materials and testing techniques. Cross-study comparability and life-cycle credibility will be further strengthened by taking end-of-life scenarios such as recycling, degradation, and environmental fate into account [29,73]. Lastly, in order to guarantee that green nanomaterials provide net sustainability advantages when scaled beyond laboratory demonstrations, circular design concepts, renewable feedstocks, low-temperature synthesis, modular replacement, and end-of-life recovery continue to be crucial [45,65,73,101].

## 6. Conclusions

The transition of Atmospheric Water Harvesting from laboratory-scale proof of concept to globally deployable infrastructure requires a fundamental paradigm shift, where scientists must stop optimizing materials based on raw gravimetric uptake at 100% relative humidity and instead focus completely on the entire kinetics and thermodynamics of the adsorption–desorption life-cycle under relevant arid conditions. The synthesized mechanistic, economic, and life-cycle framework outlined in this review establishes three paramounts, explicit design criteria that must guide the next generation of AWH nanomaterials:

Hierarchical Pore Architecture for Kinetic Dominance: Complete reliance on microporosity is seen to cause catastrophic mass transport resistance. The sorbent matrices need to be engineered to have hierarchical topologies. The Angstrom-scale micropores need to be carefully controlled to set the thermodynamic threshold for capillary condensation at low relative humidity (<30%). At the same time, the sorbent matrices need to be engineered to have an extensive and interconnected network of mesopores and macropores to function as low-resistance pathways for vapor diffusion, bypassing internal diffusion problems, eliminating hysteresis, and compressing the entire cycle time to minutes instead of hours.

Targeted Surface Chemistry and Hydrophilic Engineering: Water capture must be catalyzed by highly polar, explicitly engineered functional groups (e.g., targeted hydrogen bonding sites of pyrazole linkers in MOFs, heteroatom covalently linked groups in COFs, or oxygen-rich domains in graphene oxide). These groups must act as primary sites for water anchoring, polarizing the immediate solid-vapor interface. This must firmly anchor the initial water molecules, creating a local hydrogen bond network that must drive fast, spontaneous filling of additional water molecules entirely by fluid–fluid interactions, drastically lowering the activation energy needed to harvest vapor from hyper-arid air.

Integration of Interfacial Photothermal Regeneration: To completely disconnect AWH systems from the electrical grid and eliminate the high penalties imposed by high SEC, the bulk sorbent needs to be integrated with environmentally friendly and wide-bandgap photothermals such as reduced graphene oxide, bio-derived carbon aerogels, and biogenic metallic nanoparticles. These systems integrate the solar-to-thermal energy conversion process at the internal interface between the liquid and the solid, thus completely avoiding the tremendous sensible heat losses that result from heating the entire volume of the device and achieving complete desorption with only passive sunlight.

By adhering to these structural principles in an absolute and uncompromising way—and at the same time, prioritizing scalable and aqueous-based green synthesis protocols that are in line with the principles of minimum Cumulative Energy Demand and the elimination of the need for toxic solvent reliance—the scientific community can work towards an absolute reduction in the Levelized Cost of Water to universally accessible levels. It is in this way that the promise of Atmospheric Water Harvesting as a resilient and decentralized pillar in global water security can be fulfilled.

## Figures and Tables

**Table 1:** Key parameters governing the performance of AWH systems.

Parameter	Typical Range/Influence	Relevance	References
Ambient relative humidity (RH)	10–90%	Determines equilibrium uptake and regeneration feasibility.	[4,23,29]
Temperature (°C)	10–45	Affects the dew point and adsorption kinetics.	[4,23,29]
Sorption capacity (g H_2_O g^−1^ sorbent)	0.1–3.0	Higher capacity improves water yield per mass of material under specific RH conditions.	[26,30]
Specific energy consumption (SEC)	~0.2–1.0 kWh L^−1^ *	Indicates overall thermal efficiency and regeneration burden.	[31,32]
Regeneration enthalpy (kJ mol^−1^ H_2_O)	40–70	Energy required for desorption.	[33]
Cycling stability (cycles to >90% capacity)	100–500	Reflects durability under humid–dry cycling.	[28,34]

* Reported SEC values correspond primarily to favorable humidity and operating conditions; at low relative humidity, specific energy consumption can increase substantially due to reduced water yield and higher regeneration demand.

**Table 2:** Representative water treatment applications of green nanomaterials and key performance attributes relevant to durability, regeneration, and sustainability in AWH systems [49,50,51,52,53,62,63,64,65,66,67,68,69,70,71,72].

Treatment Approach	Mechanism/Key Process	Limitations	Nanotechnology-Based Enhancement	Main Benefits
Coagulation–flocculation	Aggregation and sedimentation of colloids using alum, Fe salts	High sludge volume; chemical use	Magnetic and photocatalytic nanoparticles (Fe_3_O_4_, TiO_2_)	Easier separation, reusability, and reduced sludge
Adsorption	Physical/chemical capture on activated carbon or zeolites	Limited surface area, costly regeneration	Green-synthesized biochar, SiO_2_, ZnO nanoadsorbents	High surface area, renewable feedstocks
Filtration/Membranes	Pressure-driven separation (micro-, ultra-, nano-filtration)	Fouling and high energy demand	Nanocomposite and antifouling membranes (TiO_2_, GO, CNF)	Lower fouling, longer lifetime
Biological treatment	Microbial degradation of organic matter	Sensitive to temperature and toxins	Nano-bio hybrids (nZVI, biochar composites)	Enhanced microbial activity, stable performance
Photocatalysis/Advanced oxidation	Degradation via radicals (UV/H_2_O_2_, TiO_2_ catalysts)	High energy cost	Green TiO_2_, ZnO, or AgNP composites	Solar activation, self-cleaning capacity

**Table 3:** (**a**). Representative examples of green and conventional synthesis pathways for AWH materials, illustrating differences in processing conditions and solvent systems reported in the literature [11,30,47,48,50,51,53,63,70,75]. (**b**). Qualitative comparison of reported energy requirements, solvent-related impacts, and relative carbon footprint indicators for green versus conventional synthesis routes of representative AWH materials. Values are indicative of trends reported under heterogeneous experimental conditions and do not represent standardized life-cycle assessments.

(**a**)						
**Material**	**Green Synthesis Route**			**Conventional Route**	**Solvent/Medium**	**References**
ZnO nanoparticles	Plant-extract-mediated biosynthesis (<80 °C; e.g., Moringa, tea polyphenols)			Chemical precipitation using NaOH/ethanol reflux	Aqueous bio-extract	[47,51]
TiO_2_ nanostructures	Sol–gel in water/ethanol with citric acid surfactant			Sol–gel using isopropoxide in DMF	Water/ethanol	[63]
SiO_2_ aerogel	Rice-husk ash or agricultural silica via acid leaching			Tetraethyl orthosilicate hydrolysis in ethanol	Water	[50,53]
Fe_2_O_3_ nanoparticles	Biogenic synthesis using microbial or leaf extracts			Chemical coprecipitation (FeCl_2_/FeCl_3_)	Aqueous	[48,75]
MOF-801/MOF-303	Water-based solvothermal at ambient pressure			DMF solvothermal synthesis	Water	[30,70]
CaO–SiO_2_ composite	Biomass-derived calcination routes			Solid-state reaction from CaCO_3_ and quartz	Biomass feed	[11]
(**b**)						
**Material**	**Energy Requirement**	**Toxic By-products**	Relative CO_2_ Footprint *	**Remarks (AWH Relevance)**		**References**
ZnO nanoparticles	Low (<80 °C)	Minimal	↓ 60%	Hydrophilic and antimicrobial surfaces improve hygienic AWH operation		[47,51]
TiO_2_ nanostructures	Moderate (<120 °C)	None	↓ 45%	Photocatalytic self-cleaning surface enhances condensation efficiency		[63]
SiO_2_ aerogel	Moderate	Ethanol waste	↓ 30%	Mesoporosity promotes capillary condensation at low RH		[50,53]
Fe_2_O_3_ nanoparticles	Low (<90 °C)	Trace salts	↓ 40%	Magnetic heat-transfer aid supports efficient desorption		[48,75]
MOF-801/MOF-303	Low (<100 °C)	None	↓ 50%	High water uptake at 10–40% RH; scalable and low-impact for AWH		[30,70]
CaO–SiO_2_ composite	Moderate (600–700 °C)	CO_2_	↓ 25%	Structural matrix for hybrid sorbents and coatings		[11]

* Arrows means percentage reduction. Percentage reductions and qualitative descriptors are derived from reported trends in the literature and depend on synthesis scale, system boundaries, and methodological assumptions. Direct numerical comparison across studies should therefore be interpreted with caution.

**Table 4:** Field performance metrics of representative nano-enabled AWH systems.

Nano-Enabled AWH System	Typical Operating Conditions	Reported Water-Harvesting Performance *	Sustainability-Enabling Component(s)	Primary Advantages	Identified Limitations	References
TiO_2_/silica biphilic fog-collection mesh	Fog tunnel (25 °C, 75–85% RH); coastal field deployment (Namibia)	≈2× enhancement relative to fully hydrophilic mesh (Demonstrated long-term outdoor robustness)	Passive surface structuring; no active energy input	Electricity-free operation; scalable mesh architecture	Gradual UV-induced wettability degradation	[72]
MOF-801 embedded in chitosan–PVA monolithic sheet (roll-to-roll)	Laboratory (30% RH, 25 °C); rooftop trials (Madrid, 15–50% RH)	1.0–1.2 g H_2_O g^−1^ in 15–25 min; ≥85% desorption in 10–15 min; <3% mass loss after 30 days	Chitosan biopolymer scaffold; low-temperature regeneration	Fast sorption kinetics; dust-free handling; mechanical robustness	Polymer dehydration under prolonged heat exposure	[70]
Sodium-alginate/CNT/MgCl_2_ composite aerogel (3 mm slab)	20–95% RH; 1-sun solar irradiation	0.27 → 5.4 g H_2_O g^−1^ (20 → 95% RH); ≈10 L kg^−1^ day^−1^ (12 cycles)	Alginate from seaweed; solar-driven regeneration	Wide-humidity operability; fully passive regeneration	CNT cost; salt migration under mechanical stress	[76]
Structured hygroscopic lattice/aerogel (salt-confined composite)	Outdoor testing (≈20% RH, sunlight)	Up to ≈0.6–0.7 L kg^−1^ day^−1^ (representative reported outdoor productivity); cycle times on the order of tens of minutes	Polymer hydrogel matrix; salt confinement; low-temperature solar regeneration	All-weather operation; high water uptake; passive regeneration capability	Salt leakage risk; long-term mechanical stability under cycling	[77]
Passive MOF-801 powder-bed “desert box.”	Mojave Desert field tests (7–25% RH)	0.6–0.8 L kg^−1^ day^−1^ (field); up to 2.8 L kg^−1^ day^−1^ (lab at 20% RH)	Passive solar heating; no moving parts	Demonstrated arid-zone functionality	Powder attrition; condenser fouling	[28]
Adaptive MOF-801 harvester with sensor-controlled airflow	Desert deployment (17–32% RH)	About 3.5 L·kg^−1^·day^−1^; 1.7–5.3 kWh L^−1^	Operational optimization (not material-based)	High yield through dynamic cycling	Added energy demand; system complexity	[68]

* Reported values are reproduced directly from the cited sources and expressed using the performance metrics employed in the original studies (mass-normalized, area-normalized, or relative enhancement). Values are therefore not directly comparable without accounting for operating conditions, cycling protocol, and normalization basis.

## Data Availability

Data supporting this study are available from the corresponding authors upon reasonable request.

## References

[1] Sachs J. (2022). Sustainable Development Report 2022.

[2] Ferroukhi R., Nagpal D., Lopez-Pena A., Hodges T., Mohtar R.H., Daher B., Mohtar S., Keulertz M. (2015). Renewable Energy in the Water, Energy & Food Nexus.

[3] Swatuk L.A. (2021). Global water crises and challenges for water security. Handbook of Security and the Environment.

[4] Nikkhah H., Azmi W.M.B.W., Nikkhah A., Najafi A.M., Babaei M.M., Fen C.S., Nouri A., Mohammad A.W., Lun A.W., Yong N.L. (2023). A comprehensive review on atmospheric water harvesting technologies: From thermodynamic concepts to mechanism and process development. J. Water Process Eng..

[5] Bai S., Tian Y., Zeng Y., Chao L., Pan A., Ho T., Chen S., Shang J., Tso C. (2023). Adsorption-based atmospheric water harvesting by passive radiative condensers for continuous decentralized water production. Appl. Therm. Eng..

[6] Sadek S., Deng S., Zhao J., Zayed M.E. (2022). Solar-powered adsorption-based atmospheric water harvesting systems: Principles, materials, performance analysis, and configurations. Sustain. Energy Technol. Assess..

[7] Lee Y., Fan S., Yang S. (2025). Nature-Inspired Design Strategies for Efficient Atmospheric Water Harvesting. Adv. Mater..

[8] Rao A.K., Fix A.J., Yang Y.C., Warsinger D.M. (2022). Thermodynamic limits of atmospheric water harvesting. Energy Environ. Sci..

[9] Kwan T.H., Yuan S., Shen Y., Pei G. (2022). Comparative meta-analysis of desalination and atmospheric water harvesting technologies based on the minimum energy of separation. Energy Rep..

[10] Thakur A.K., Hazra S.K., Saleque A.M., Bhattarai S., Hwang J.-Y., Ahamed M.S. (2024). Toward sustainable water solutions: A review of nanomaterials for solar-driven water harvesting. ACS EST Water.

[11] Feng A., Akther N., Duan X., Peng S., Onggowarsito C., Mao S., Fu Q., Kolev S.D. (2022). Recent development of atmospheric water harvesting materials: A review. ACS Mater. Au.

[12] Mardani M., Rakhshandehroo G., Zerafat M. (2025). Moisture Harvesting Efficacy Utilizing Flower-Like ZnO Nanostructures Coated on Cotton Fabrics. Iran. J. Sci. Technol. Trans. Civ. Eng..

[13] Kim H., Rao S.R., Kapustin E.A., Zhao L., Yang S., Yaghi O.M., Wang E.N. (2018). Adsorption-based atmospheric water harvesting device for arid climates. Nat. Commun..

[14] Huang X., Qin Q., Ma Q., Wang B. (2022). Atmospheric water harvesting with metal-organic frameworks and their composites: From materials to devices. Water.

[15] Zhang S., Xu S., Lei R., Pan Y., Ma T., Zhang Z., Liu C., Zhang Z. (2023). Core-shell-embedded Mesoporous Silica Capsules for Atmospheric Water Harvesting. J. Wuhan Univ. Technol.-Mater. Sci. Ed..

[16] Shi Y., Feng A., Mao S., Onggowarsito C., Zhang X.S., Guo W., Fu Q. (2024). Hydrogels in solar-driven water and energy production: Recent advances and future perspectives. Chem. Eng. J..

[17] Shi L., Kirlikovali K.O., Chen Z., Farha O.K. (2024). Metal-organic frameworks for water vapor adsorption. Chem.

[18] Severino M.I., Freitas C., Pimenta V., Nouar F., Pinto M.L., Serre C. (2025). Cost estimation of the production of MIL-100 (Fe) at industrial scale from two upscaled sustainable synthesis routes. Ind. Eng. Chem. Res..

[19] Oktor K., Dhuol M.G.R., Kalkan M.E. (2024). Fog harvesting: An effective solution to the water scarcity problem. Sak. Univ. J. Sci..

[20] Aqualonis GmbH Fog Harvesting. https://www.aqualonis.com.

[21] Sustainable Water Solutions. https://fogquest.org/.

[22] Warka Village: Guardian of the Forest. https://www.warkawater.org/.

[23] Corraide da Silva L., Oliveira Filho D., Acioli Imbuzeiro H.M., Barros Monteiro P.M. (2022). Analysis of different condensing surfaces for dew harvesting. Water Supply.

[24] Xia X., Li S. (2023). Improved adsorption cooling performance of MIL-101 (Cr)/GO composites by tuning the water adsorption rate. Sustain. Energy Fuels.

[25] Terzis A., Ramachandran A., Wang K., Asheghi M., Goodson K.E., Santiago J.G. (2020). High-frequency water vapor sorption cycling using fluidization of metal-organic frameworks. Cell Rep. Phys. Sci..

[26] Hanikel N., Pei X., Chheda S., Lyu H., Jeong W., Sauer J., Gagliardi L., Yaghi O.M. (2021). Evolution of water structures in metal-organic frameworks for improved atmospheric water harvesting. Science.

[27] Parida V.K., Das S., Maity S., Mahanty A., Datta D., Pradhan A. (2025). Green Synthesis of Nanocatalysts and Nanomaterials for Effluent Treatment. Sustainable Effluent Treatment and Resource Recovery, Volume 1.

[28] Kim H., Yang S., Rao S.R., Narayanan S., Kapustin E.A., Furukawa H., Umans A.S., Yaghi O.M., Wang E.N. (2017). Water harvesting from air with metal-organic frameworks powered by natural sunlight. Science.

[29] Wang J., Hua L., Li C., Wang R. (2022). Atmospheric water harvesting: Critical metrics and challenges. Energy Environ. Sci..

[30] Aghajani Hashjin M., Zarshad S., Motejadded Emrooz H.B., Sadeghzadeh S. (2023). Enhanced atmospheric water harvesting efficiency through green-synthesized MOF-801: A comparative study with solvothermal synthesis. Sci. Rep..

[31] Gayoso N., Moylan E., Noha W., Wang J., Mulchandani A. (2024). Techno-economic analysis of atmospheric water harvesting across climates. ACS EST Eng..

[32] Potyka J., Dalibard A., Tovar G. (2024). Energetic analysis and economic viability of active atmospheric water generation technologies. Discov. Appl. Sci..

[33] El-Sharkawy I.I., Haridy S., Hassan M., Radwan A., Abd-Elhady M.M. (2024). Optimization of atmospheric water harvesting cycles for sustainable water supply in arid regions. Int. J. Thermofluids.

[34] Song W., Zheng Z., Alawadhi A.H., Yaghi O.M. (2023). MOF water harvester produces water from Death Valley desert air in ambient sunlight. Nat. Water.

[35] Bagheri F. (2018). Performance investigation of atmospheric water harvesting systems. Water Resour. Ind..

[36] Watergen Ltd. Technology. https://www.watergen.com/technology/.

[37] Kiyabu S., Shkatulov A., Ahmed A., Greene S.M., Huinink H.P., Siegel D.J. (2026). Materials for Thermochemical Energy Storage and Conversion: Attributes for Low-Temperature Applications. Mater. Horiz..

[38] SOURCE Global How It Works. https://source.co/pages/how-it-works.

[39] Lin H., Song Y., Ding Z., Sui Y., Sui Z., Li F., Zhu J., Wu W. (2025). Multi-stage power-to-water battery synergizes flexible energy storage and efficient atmospheric water harvesting. Nat. Commun..

[40] Lei X., Shao C., Shou X., Shi K., Shi L., Zhao Y. (2021). Porous hydrogel arrays for hepatoma cell spheroid formation and drug resistance investigation. Bio-Des. Manuf..

[41] Garba Z.N., Ratanatamskul C. (2025). Kinetics, adsorption mechanism, and economic viability of an eco-friendly amorphous carbon thin-film adsorbent synthesized from agricultural waste for removal of 2,4-dichlorophenol and 2,4,6-trichlorophenol in water environment. Case Stud. Chem. Environ. Eng..

[42] Wang Y., Danook S.H., AL-bonsrulah H.A., Veeman D., Wang F. (2022). A recent and systematic review on water extraction from the atmosphere for arid zones. Energies.

[43] Tene T., Tubon Usca G., Guevara M., Molina R., Veltri F., Arias M., Caputi L.S., Vacacela Gomez C. (2020). Toward large-scale production of oxidized graphene. Nanomaterials.

[44] Pathania S., Jyoti A., Rathour A. (2025). Understanding enzyme immobilization: Methods, technologies, and applications. Enzyme Immobilization with Nanomaterials: Applications and Challenges.

[45] Elzein B. (2024). Nano Revolution: “Tiny tech, big impact: How nanotechnology is driving SDGs progress”. Heliyon.

[46] Pechyen C., Tangnorawich B., Toommee S., Marks R., Parcharoen Y. (2024). Green synthesis of metal nanoparticles, characterization, and biosensing applications. Sens. Int..

[47] Gupta S., Choudhary D.K., Sundaram S. (2025). Green synthesis and characterization of silver nanoparticles using Citrus sinensis (Orange peel) extract and their antidiabetic, antioxidant, antimicrobial and anticancer activity. Waste Biomass Valorization.

[48] Abdullah J.A.A., Eddine L.S., Abderrhmane B., Alonso-González M., Guerrero A., Romero A. (2020). Green synthesis and characterization of iron oxide nanoparticles by pheonix dactylifera leaf extract and evaluation of their antioxidant activity. Sustain. Chem. Pharm..

[49] Zou Z., Luo X., Wang L., Zhang Y., Xu Z., Jiang C. (2021). Highly mesoporous carbons derived from corn silks as high performance electrode materials of supercapacitors and zinc ion capacitors. J. Energy Storage.

[50] Monica M., Irine J., Jayasree R. (2024). Eco-Friendly Synthesis of Mesoporous Silica Nanoparticles from Banana Peel Waste: A Comprehensive Study on Sustainable Waste Utilization and Advanced Material Development. Res. Sq..

[51] Jovanović D., Bognár S., Despotović V., Finčur N., Jakšić S., Putnik P., Deák C., Kozma G., Kordić B., Šojić Merkulov D. (2024). Banana peel extract-derived ZnO nanopowder: Transforming solar water purification for safer agri-food production. Foods.

[52] Wagh S.S., Kadam V.S., Jagtap C.V., Salunkhe D.B., Patil R.S., Pathan H.M., Patole S.P. (2023). Comparative studies on synthesis, characterization and photocatalytic activity of Ag doped ZnO nanoparticles. ACS Omega.

[53] Ge L., Feng Y., Xue Y., Dai Y., Wang R., Ge T. (2023). Mesoporous Silica-Guided Synthesis of Metal–Organic Framework with Enhanced Water Adsorption Capacity for Smart Indoor Humidity Regulation. Small Struct..

[54] Liao Y., Ma X., Zou J., Zhao M., Chen D., Xu D., Yuan B. (2025). Preparation and adsorption properties of microsphere geopolymers derived from calcium carbide slag and fly ash. Sci. Rep..

[55] Shen X., Ou R., Lu Y., Yuan A., Liu J., Gu J., Hu X., Yang Z., Yang F. (2020). Record-high capture of volatile benzene and toluene enabled by activator implant-optimized banana peel-derived engineering carbonaceous adsorbents. Environ. Int..

[56] Tymoshok N., Demchenko O., Kharchuk M., Bityutskyy V., Tsekhmistrenko O., Tsekhmistrenko S. (2025). Study of genus *Bacillus* (*B. clausii*) probiotic bacteria regarding the biogenic extracellular synthesis of selenium nanoparticles. Mikrobiolohichnyi Zhurnal.

[57] Salman M., Ismail M., Ullah B., Khan M.M., Hussein M., Khan J.U., Ahmad B., Bashar N.U., Baseer A., Munir S. (2023). The role of *Bacillus* species in the synthesis of metal and metal oxide nanoparticles and their biomedical applications: A mini review. Nanomed. J..

[58] Slavin Y.N., Bach H. (2022). Mechanisms of antifungal properties of metal nanoparticles. Nanomaterials.

[59] Bag D.S.S., Bora A., Golder A., Raina K., Haridhasapavalan K.K., Thummer R.P. (2022). Gelatin-Pva-AgNPs Triad Composite as Wound Healing Hydrogel with Wounded Skin Surface Protective Efficiency. https://ssrn.com/abstract=4219683.

[60] Pati P., McGinnis S., Vikesland P.J. (2014). Life cycle assessment of “green” nanoparticle synthesis methods. Environ. Eng. Sci..

[61] Al-Sadeq N., Perez-Puyana V.M., Romero A., Abdullah J.A.A. (2025). Enhancing Atmospheric Water Harvesting Applications through the Integration of Green Silica and Zinc Oxide Nanoparticles into Chitosan Biopolymer. Res. Sq..

[62] Thamarai P., Kamalesh R., Saravanan A., Swaminaathan P., Deivayanai V. (2024). Emerging trends and promising prospects in nanotechnology for improved remediation of wastewater contaminants: Present and future outlooks. Environ. Nanotechnol. Monit. Manag..

[63] Nguyen B.C., Truong T.M., Nguyen N.T., Dinh D.N., Hollmann D., Nguyen M.N. (2024). Advanced cellulose-based hydrogel TiO_2_ catalyst composites for efficient photocatalytic degradation of organic dye methylene blue. Sci. Rep..

[64] Osman A.I., Chen Z., Elgarahy A.M., Farghali M., Mohamed I.M., Priya A., Hawash H.B., Yap P.S. (2024). Membrane technology for energy saving: Principles, techniques, applications, challenges, and prospects. Adv. Energy Sustain. Res..

[65] Saud A., Gupta S., Allal A., Preud’Homme H., Shomar B., Zaidi S.J. (2024). Progress in the sustainable development of biobased (nano) materials for application in water treatment technologies. ACS Omega.

[66] Wang H., Wang T., Xue G., Zhao J., Ma W., Qian Y., Wu M., Zhang Z., Gao P., Su C. (2021). Key technologies and equipment for contaminated surface/groundwater environment in the rural river network area of China: Integrated remediation. Environ. Sci. Eur..

[67] Huang J., Tian W., Liu C., Wang S., Xie L. (2025). MOF-derived composite enables efficient reduction and rapid capture for gold recovery from e-waste leachates. Sep. Purif. Technol..

[68] Chekli L., Bayatsarmadi B., Sekine R., Sarkar B., Shen A.M., Scheckel K.G., Skinner W., Naidu R., Shon H.K., Lombi E. (2016). Analytical characterisation of nanoscale zero-valent iron: A methodological review. Anal. Chim. Acta.

[69] European Water Association Pilot Demonstrations of Bio-Derived Cellulose Aerogels for Drinking-Water Sand-Filter Retrofits. https://www.ewa-online.eu/.

[70] Riva L., Dotti A., Iucci G., Venditti I., Meneghini C., Corsi I., Khalakhan I., Nicastro G., Punta C., Battocchio C. (2024). Silver nanoparticles supported onto TEMPO-oxidized cellulose nanofibers for promoting Cd^2+^ cation adsorption. ACS Appl. Nano Mater..

[71] Tian G., Fu C., Guo Z. (2024). Biomimetic fog collector with hybrid and gradient wettabilities. ACS Appl. Mater. Interfaces.

[72] Kumar N. (2024). Microporous and mesoporous materials for catalytic applications. Catalysts.

[73] Rabiee N., Sharma R., Foorginezhad S., Jouyandeh M., Asadnia M., Rabiee M., Akhavan O., Lima E.C., Formela K., Ashrafizadeh M. (2023). Green and sustainable membranes: A review. Environ. Res..

[74] Ho T.G.-T., Truong D.P.T., Nguyen H.B., Do B.L., Dinh T.A., Ton-That P., Van Nguyen T.T., Truong T.B.T., Huynh K.P.H., Tri N. (2023). Green synthesized nano-silver/cellulose aerogel as a robust active and recyclable catalyst towards nitrophenol hydrogenation. Chem. Eng. J..

[75] Yoon Y., Truong P.L., Lee D., Ko S.H. (2021). Metal-oxide nanomaterials synthesis and applications in flexible and wearable sensors. ACS Nanosci. Au.

[76] Zhu P., Yu Z., Sun H., Zheng D., Zheng Y., Qian Y., Wei Y., Lee J., Srebnik S., Chen W. (2024). 3D printed cellulose nanofiber aerogel scaffold with hierarchical porous structures for fast solar-driven atmospheric water harvesting. Adv. Mater..

[77] Zhao F., Zhou X., Liu Y., Shi Y., Dai Y., Yu G. (2019). Super moisture-absorbent gels for all-weather atmospheric water harvesting. Adv. Mater..

[78] Fu C., He Y., Yu A., Tian G., Zhan D., Zhang H., Guo Z. (2024). Vertical macroporous chitosan aerogel adsorbents for simple and efficient enhancement of atmospheric water harvesting and air dehumidification. J. Mater. Chem. A.

[79] Ren X., Sui X., Karton A., Nishina Y., Lin T., Asanoma D., Owens L., Ji D., Wen X., Quintano V. (2025). Supporting information: Synergetic hydrogen-bond network of functionalized graphene and cations for enhanced atmospheric water capture. Proc. Natl. Acad. Sci. USA.

[80] Xia X., Liu B., Zhao B., Xia Z., Li S. (2023). Enhanced water adsorption of MIL-101 (Cr) by metal-organic polyhedral encapsulation for adsorption cooling. Nanomaterials.

[81] Hassan A.A., Ezzeddine M., Kordy M.G., Awad M.M. (2023). Techno-economic assessment of atmospheric water harvesting (AWH) technologies. Atmospheric Water Harvesting Development and Challenges.

[82] Anjali C., Renuka N.K. (2022). Atmospheric water harvesting: Prospectus on graphene-based materials. J. Mater. Res..

[83] Huang Y., Wang C., Shao C., Wang B., Chen N., Jin H., Cheng H., Qu L. (2021). Graphene oxide assemblies for sustainable clean-water harvesting and green-electricity generation. Acc. Mater. Res..

[84] Rodríguez-Rojas M.d.P., Bustos-Terrones V., Díaz-Cárdenas M.Y., Vázquez-Vélez E., Martínez H. (2024). Life cycle assessment of green synthesis of TiO_2_ nanoparticles vs. chemical synthesis. Sustainability.

[85] Hanikel N., Prévot M.S., Fathieh F., Kapustin E.A., Lyu H., Wang H., Diercks N.J., Glover T.G., Yaghi O.M. (2019). Rapid cycling and exceptional yield in a metal-organic framework water harvester. ACS Cent. Sci..

[86] Tao Y., Zhu B., Zhu D., Li H. (2024). Thermoelectrically regulating heat flux in metal−organic framework monoliths for high-yield atmospheric water harvesting in arid regions. Ind. Eng. Chem. Res..

[87] Peng R., Bai Y., Xie Y., Zhu D. (2025). cis/trans Octahedral configuration induced topologically different MOFs: Syntheses, structures, and Hirshfeld surface analyses. Chin. J. Inorg. Chem..

[88] Peng R., Xie Y., Yuan S., Shen R., Zhu D. Metal-Organic Frameworks (2014–2024): A decade pursuit for top performance. Acta Phys.-Chim. Sin..

[89] He Y., Ran J., Gao X., Ding J., Templeton M.R., Peng C., Chu W. (2025). Covalent organic frameworks enable efficient atmospheric water harvesting in arid climates. Environ. Sci. Water Res. Technol..

[90] Nguyen H.L., Hanikel N., Lyle S.J., Zhu C., Proserpio D.M., Yaghi O.M. (2020). A porous covalent organic framework with voided square grid topology for atmospheric water harvesting. J. Am. Chem. Soc..

[91] Sonji G., Sonji N., El Katerji A., Rahal M. (2025). Green Aerogels for Atmospheric Water Harvesting: A PRISMA-Guided Systematic Review of Bio-Derived Materials and Pathways to 2035. Polymers.

[92] Zhang X., Qu H., Li X., Zhang L., Zhang Y., Yang J., Zhou M., Suresh L., Liu S., Tan S.C. (2024). Autonomous atmospheric water harvesting over a wide RH range enabled by super hygroscopic composite aerogels. Adv. Mater..

[93] Feng Y., Ge L., Zhao Y., Li Q., Wang R., Ge T. (2024). Active MOF water harvester with extraordinary productivity enabled by cooling-enhanced sorption. Energy Environ. Sci..

[94] Gao C., Yu D., Zhu L., Wei H., Zhang L., Zhou M., Zhang T., Tian B., Wang J., Hou Y. (2025). Robust Bioinspired Microcellular and Micro-Nanochannel Photothermal Aerogels for High-Efficiency Atmospheric Water Harvesting. ACS Nano.

[95] Dong S., Li G., Jin S., Hu H., Ye G. (2025). Recent Advances and Retrospective Review in Bioinspired Structures for Fog Water Collection. Biomimetics.

[96] Flemming H.-C., Wingender J., Szewzyk U., Steinberg P., Rice S.A., Kjelleberg S. (2016). Biofilms: An emergent form of bacterial life. Nat. Rev. Microbiol..

[97] Gizer G., Önal U., Ram M., Şahiner N. (2023). Biofouling and mitigation methods: A review. Biointerface Res. Appl. Chem..

[98] Le-Clech P., Chen V., Fane T.A. (2006). Fouling in membrane bioreactors used in wastewater treatment. J. Membr. Sci..

[99] Zodrow K., Brunet L., Mahendra S., Li D., Zhang A., Li Q., Alvarez P.J. (2009). Polysulfone ultrafiltration membranes impregnated with silver nanoparticles show improved biofouling resistance and virus removal. Water Res..

[100] Rasmussen K., Schoonjans R., Jantunen P., Rauscher H., Szpunar J., Jiménez-Lamana J. (2022). European Union Legislation Addressing Environment, Health and Safety Aspects of Nanomaterials. Environmental Nanopollutants: Sources, Occurrence, Analysis and Fate.

[101] Pathak J., Xavier K.M., Ngasotter S., Goswami A., Hazarika U., Saikia R. (2025). Sustainable nanotechnology for green environment. Waste Derived Carbon Nanomaterials.

[102] Sessa A., Rossi E., Prete P., Passarini F., Itatani M., Rossi F., Lagzi I., Lo Nostro P., Cespi D., Cucciniello R. (2025). Life Cycle Assessment of Solvothermal Zeolitic Imidazolate Framework-8 Synthesis: Is the Substitution of N, N-Dimethylformamide with Glycerol Carbonate Environmentally Sustainable?. ChemSusChem.

[103] Zheng Z., Alawadhi A.H., Yaghi O.M. (2023). Green synthesis and scale-up of MOFs for water harvesting from air. Mol. Front. J..

[104] Vardhan H., Rummer G., Deng A., Ma S. (2023). Large-scale synthesis of covalent organic frameworks: Challenges and opportunities. Membranes.

[105] Zhu C., Pang S., Chen Z., Bi L., Wang S., Liang C., Qin C. (2022). Synthesis of covalent organic frameworks (COFs)-nanocellulose composite and its thermal degradation studied by TGA/FTIR. Polymers.

